# Knockdown of ATF3 suppresses the progression of ischemic stroke through inhibiting ferroptosis

**DOI:** 10.3389/fnmol.2022.1079338

**Published:** 2023-01-18

**Authors:** Jin Ye, Fan Zhang, Bin Li, Qing Liu, Guoyong Zeng

**Affiliations:** ^1^The Office of Stroke Screening and Prevention, Ganzhou People's Hospital, Ganzhou, Jiangxi, China; ^2^Department of Neurology, Ganzhou People's Hospital, Ganzhou, Jiangxi, China

**Keywords:** ischemic stroke, ferroptosis, ATF3, differentially expressed genes, neurology

## Abstract

**Objective:**

Current therapies towards ischemic stroke (IS) are still not satisfied, and alternative strategies targeting ferroptosis may be another choice. The purpose of this study is to screen potential ferroptosis-related genes involving in IS.

**Methods:**

A rat model of IS was established *via* middle cerebral artery occlusion. Differentially expressed genes (DEGs) were screened from the model rats through transcriptional sequencing. Among the isolated DEGs, the expression of several attractive DEGs relating with ischemic injury was confirmed by qRT-PCR. Then, ATF3 relating with both IS and ferroptosis was selected a candidate gene for functional assays. After knockdown of ATF3 in the model rats, the infarction, histopathology, apoptosis, and ferroptosis in brain tissues were evaluated.

**Results:**

IS model was successfully established in rats, exhibiting the emergence of infarction area, histopathological injury, and enhanced cell apoptosis. Total 699 up-regulated DEGs and 461 down-regulated DEGs were screened from the model rats. qRT-PCR verified the up-regulation of Hspa1b, Tfpi2, Ptx3, and Atf3, and the down-regulation of Smyd1 and Tacr2 in the Model group compared with those in the Sham group. It is noteworthy that knockdown of ATF3 decreased the infarction area, relieved histopathological injury, weakened apoptosis, and inhibited ferroptosis in the model rats.

**Conclusion:**

Several candidate genes in relation with IS were revealed. More importantly, knockdown of ATF3 may relieve IS through inhibiting ferroptosis.

## Introduction

Ischemic stroke (IS) is an acute cerebrovascular disease results from a sudden blockage of blood supply in the brain (Khoshnam et al., 2017). Because IS can directly induce neurological damage, it remains the leading cause of human disability and mortality in the world (Uzdensky, 2019). Until now, the development of pharmacological and mechanical thrombolysis has made certain progress in helping patients recover from IS (Paul and Candelario-Jalil, 2021). However, neuroprotective intervention is still needed because blood flow recanalization is usually accompanied with ischemia/reperfusion injury (Tuo et al., 2022). Therefore, more effective therapeutic strategies in ameliorating the impaired brain function in patients with IS are urgently needed.

The pathogenesis of IS is closely associated with a complex and interconnected cascade of cellular and molecular events, mainly including excitotoxicity, energy metabolism disorder, calcium overload, oxidative stress, neuroinflammation, autophagy, and apoptosis (Candelario-Jalil, 2009; Zhao et al., 2017). It is noteworthy that ferroptosis also plays a key role in IS. Ferroptosis is an iron-dependent form of programmed cell death characterized by the accumulation of lipid peroxides (Pan et al., 2021). Evidence has determined that stroke can trigger ferroptosis through inducing iron overload and lipid metabolism (Xu et al., 2022). Correspondingly, inhibition of ferroptosis is a promising target to relieve the neurological damage of IS (Liu et al., 2020). In recent years, many molecular targets show therapeutic potential against IS through regulating ferroptosis, such as ACSL4 (Cui et al., 2021), NCOA4 (Li et al., 2021), miRNA-24a (Zhang et al., 2022), SATB1 (Zhan et al., 2022), cPKCγ (Wei et al., 2022), etc. However, there are still massive potential targets that need to be explored.

Based on sequencing profiles, bioinformatics analysis has been widely applied to screen potential molecular targets for IS (Zhai et al., 2017; Zhou et al., 2019; Xie et al., 2020; Chen et al., 2021; Liu C. et al., 2022). Chen et al. (2021) have determined that MAP1LC3B, PTGS2, and TLR4 are ferroptosis-related biomarkers for IS. Liu C. et al. (2022) have revealed four ferroptosis-related therapeutic targets for IS, including HMOX1, CYBB, STAT3, and TLR4. In order to reveal more effective targets for IS, a transcriptional sequencing was also performed in this study. Among diverse differentially expressed genes (DEGs), ATF3 was selected a candidate gene due to two reasons. First, the up-regulation of ATF3 is also identified in peripheral blood mononuclear cells of IS patients (Zhai et al., 2017; Li S. et al., 2022). However, the detail function of ATF3 on IS is rarely reported. Second, ATF3 acts an important regulator in ferroptosis. For examples, ATF3 sensitizes gastric cancer cells to cisplatin through inducing ferroptosis (Fu et al., 2021); ATF3 promotes brucine-triggered ferroptosis in glioma cells through increasing H_2_O_2_ and iron (Lu et al., 2021). Therefore, we suspect that whether ATF3 is involved in the progression of IS *via* regulating ferroptosis.

In this study, a rat model of IS was established and then used to reveal the DEGs *via* transcriptional sequencing. Some important DEGs involving ischemic injury were identified, among which ATF3 was selected for functional assay due to its important role in ferroptosis. Our results may provide new insights into the underlying molecular mechanisms of IS and reveal potential therapeutic targets.

## Materials and methods

### Establishment of is model in rats

Animal experiments were approved by the Local Ethical Committee in accordance with the Guide for the Care and Use of Laboratory Animals. Wild-type male Sprague–Dawley rats (180–200 g weight) were provided by SiPeiFu Biotechnology (Beijing, China) and housed in pathogen-free condition. Middle cerebral artery occlusion (MCAO) was used to induce IS as previously described (Wang P. et al., 2021). Briefly, rats were anesthetized by intraperitoneal injection of pentobarbital sodium (40 mg/kg) and then fixed in a supine position to expose the neck area. An incision was made along the median neck to separate the common carotid artery (CCA), internal carotid artery, and external carotid artery (ECA) from adjacent tissues and vagus nerve. After the distal ECA was tied off and opened by arteriotomy, a nylon monofilament was inserted and advanced upwards about 1 cm past the CCA bifurcation. After 90 min of occlusion, the suture was removed for reperfusion (*N* = 6, Model group). During the surgery, all rats were maintained at 37°C to avoid death. Similar surgery without occlusion was performed in the Sham group (*N* = 6).

### Adenovirus interventions

Recombinant adenoviruses carrying shRNA-ATF3 (ad-shATF3-1 and - 2) and corresponding negative control (ad-shNC) were synthesized in RiboBio (Guangzhou, China). Rats were randomized to model, ad-shATF3-1, ad-shATF3-2, and ad-shNC groups (*N* = 6 each group). The recombinant adenoviruses (2 × 10^12^ vg) were intraparenchymal injected into rats at 1 week before MCAO. Subsequently, the MCASO or sham surgery was performed as mentioned above. After the reperfusion for 24 h, rats were finally sacrificed by intraperitoneal injection of an overdose of pentobarbital sodium (150 mg/kg), and the brain tissues were collected.

### TTC staining

The brain tissues were frozen at −20°C for 30 min and then sliced into 2 mm coronal sections. Subsequently, the sections were incubated with 2% TTC solutions (Solarbio, China) for 15 min at 37°C in the dark. After fixed in 4% paraformaldehyde for 24 h, the brain samples were observed under a stereomicroscope (Olympus, Japan). The unstained white area presented the infarcted tissues, and stained red area presented non-infarcted tissues.

### HE staining

For HE staining, the brain tissues were fixed in 4% paraformaldehyde, dehydrated in graded ethanol, paraffin-embedded, and sliced into 5 μM sections. The sections were then dewaxed in xylene, rehydrated in graded ethanol, and stained with Hematoxylin for 5 min and with Eosin for 2 min. After dehydration and vitrification, the stained sections were observed under an inverted microscope (Olympus).

### IHC staining

For IHC staining, the paraffin-embedded sections were prepared as mentioned above. After dewaxing and dehydration, the sections received 10 min of microwave irradiation in 10 mM citrate buffer for antigen retrieval. The sections were then blocked with goat serum in PBS, incubated with anti-caspase-3 (1:1000, Cell Signaling Technology, Danvers, MA, United States) overnight at 4°C, and continuous incubated with horseradish peroxidase-conjugated secondary antibody (1:3000, Cell Signaling Technology) for 1 h at 37°C. Subsequently, the sections were stained with diaminobenzidine for 10 min and counterstained with hematoxylin for 1 min. Followed by dehydration and vitrification, the stained sections were observed under an inverted microscope (Olympus).

### TUNEL assay

The TUNEL assay was carried out using a TUNEL Apoptosis Assay kit (Beyotime, Beijing, China). Briefly, the deparaffined/rehydrated tissue sections were incubated with DNase-free Proteinase K for 20 min, and then with TUNEL solution for 1 h in the dark. After counterstained with DAPI for 10 min, the apoptotic cells were observed and counted under a fluorescence microscope (Olympus).

### Transcriptome sequencing analysis

Brain tissue samples from the Sham and Model groups were used for transcriptome sequencing. Briefly, total RNAs isolated from the samples were qualified by Agilent 2,100 Bioanalyzer. mRNAs were purified from total RNAs *via* polyA-selection, and randomly fragmented into 200–300 bp using metal ion-catalyzed hydrolysis. Subsequently, cDNA library (about 450 bp) was constructed and sequenced on Illumina Novaseq 6,000 platform. DEGs (|log2FoldChange| > 1 and *p* < 0.05) were clustered by Pheatmap, and functional enriched by Gene ontology (GO) and Kyoto Encyclopedia of Genes and Genomes (KEGG) pathway analyses.

### Quantitative real-time PCR

Total RNA was extracted from the brain tissues using TRIzol reagent (Beyotime). cDNA was reverse transcribed from total RNA using FastKing RT Kit (TIANGEN, Beijing, China). Quantitative real-time PCR (qRT-PCR) was performed using SYBR Green PCR Master Mix (Bio-Rad, Hercules, CA, United States) on a LightCycler® 480 System (Roche, Basel, Switzerland) with standard procedures. Relative expression level was calculated following the 2^−ΔΔCt^ method using GAPDH as the internal control. The primer sequences were shown in Supplementary Table S1.

### Western blot

Protein samples were extracted from the brain tissues by lysing in RIPA buffer (Beyotime). The isolated proteins were separated by 10% sodium dodecyl sulfate-polyacrylamide gel electrophoresis and transferred to polyvinylidenefluoride membranes. The membranes were then blocked with 5% non-fat milk for 1 h, and incubated with primary antibody (anti-ATF-3, -GPX4, -COX2, and -GAPDH; 1:1000, Cell Signaling Technology) for 12 h at 4°C. After incubated with horseradish peroxidase-conjugated secondary antibody (anti-rabbit IgG, 1:3000, Cell Signaling Technology) for 1 h at 37°C, the protein blots were visualized using Electrochemiluminescence Kit (Beyotime) and analyzed by a Gel Imaging System (Tanon 5,200, Shanghai, China) with ImageJ software.

### Iron measurement

The iron content in brain tissues was measured using Iron Assay Kit (Sigma, St. Louis, MO, United States) in accordance with the manufacturer’s instructions. Simply, the tissue samples were homogenized on ice and centrifuged at 13,000 g for 10 min. Subsequently, the supernatants were incubated with iron detection buffer for 30 min at 25°C. The optical density at 593 nm was measured using a microplate reader. The iron content was finally calculated according to the standard curves.

### Measurements of glutathione and malondialdehyde

The GSH and MDA in brain tissues were measured using GSH assay kit (Solarbio) and MDA assay kit (Solarbio), respectively. Tissue homogenates were used for measurements strictly in accordance with the manufacturer’s instructions. The contents of GSH and MDA were finally calculated according to the standard curves.

### Statistical analysis

Data were expressed as mean ± standard deviation (SD), and statistical analyzed by GraphPad Prism 8.0 (GraphPad, San Diego, CA, United States). Student’s *t*-test was used for the comparisons between two groups. One-way ANOVA followed by Tukey’s post-hoc test was used for the comparisons among multiple groups. A *p* value less than 0.05 presented statistically significant.

## Results

### Characterization of brain tissues in is rats

**Figure 1:**
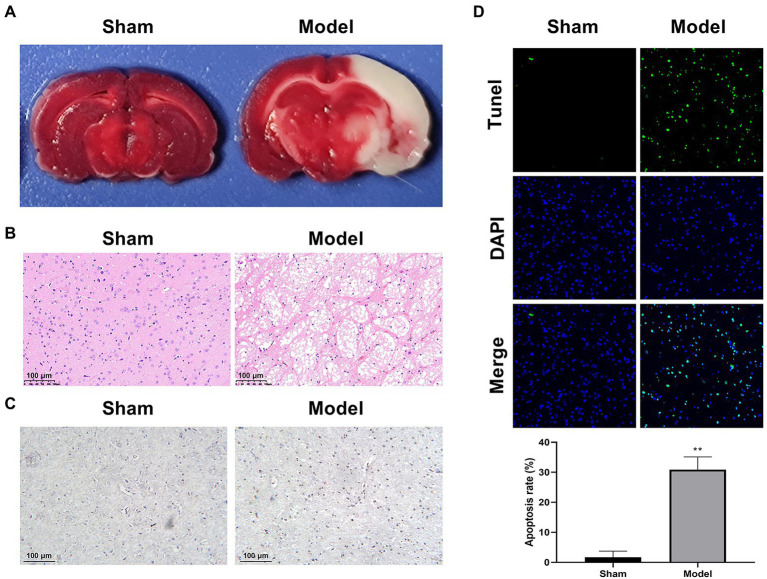
Characterization of a rat model of IS (*N* = 6 each group). **(A)** TTC staining. **(B)** HE staining. **(C)** IHC staining for caspase-3. **(D)** TUNEL staining. ^**^p < 0.01 vs. Sham.

A rat model of IS was established by MCAO. TTC staining was performed to determine the infarcted area in brain tissues, which could react with active dehydrogenases in normal tissues (red), but not with inactive dehydrogenases in infarcted tissues (white). Consistent with the expectation, the Model group showed an obvious white straining compared with the Sham group (Figure 1A). HE staining revealed interstitial edema, inflammatory cell infiltration, and disordered cell shape in the Model group in contrast to the Sham group (Figure 1B). In addition, more cells positive to caspase-3 were observed by IHC staining in the Model group than that in the Sham group, reflecting the elevated cell apoptosis in the model rats (Figure 1C). The enhanced apoptosis was further determined by TUNEL staining in the Model group when compared with the Sham group (*p* < 0.01; Figure 1D). All the results above proved the successful establishment of IS model in rats.

### Transcriptional analysis of brain tissues in is rats

**Figure 2:**
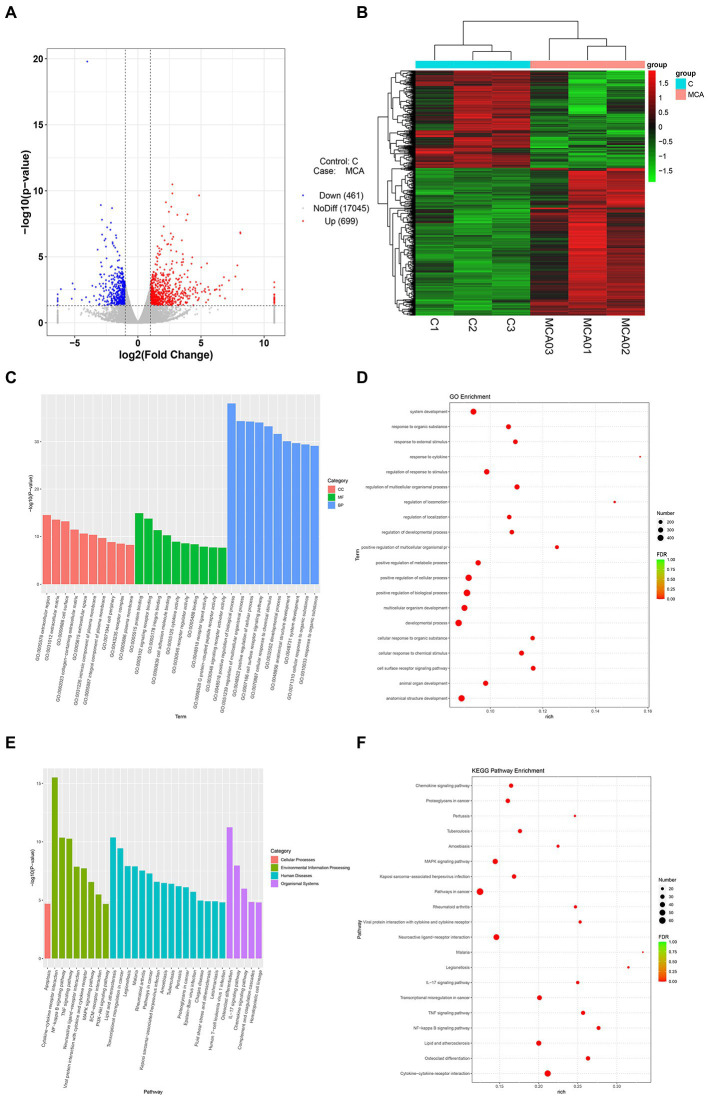
Transcriptional analysis of DEGs in brain tissues from IS rats (*N* = 6 each group). **(A,B)** Volcano plot and heatmap of DEGs between the Model (MCA01-MCA03) and the Sham groups (C1-C3). The genes with |log2FoldChange| > 1 and *p* value <0.05 defined as DEGs. **(C,D)** GO enrichment analysis of the DEGs. Column chart, top 10 GO terms with minimum *p* value in each of MF, BP, and CC were shown. MF, molecular function; BP, biological process; CC, cellular component; Bubble chart, top 20 GO terms with minimal FDR value were shown. **(E,F)** KEGG pathway analysis of the DEGs. Column chart, top 10 KEGG pathways with minimum *p* value in each category were shown. Bubble chart, top 20 KEGG pathways with minimal FDR value were shown.

Following the characterization of the rat model of IS, we focused on the DEGs at the transcriptional level. Total 1,160 DEGs were identified by transcriptome sequencing between the Model and Sham groups, including 699 up-regulated DEGs and 461 down-regulated DEGs (Figures 2A,B). The top 30 up-regulated genes and top 10 down-regulated genes were shown in Table 1. Subsequently, the isolated DEGs were enriched by GO and KEGG analyses. GO functional analysis showed that the DEGs were mainly enriched in system development, regulation of multicellular organismal process, positive regulation of biological process, and anatomical structure development, etc (Figures 2C,D). KEGG pathway analysis revealed the enrichment of DEGs in cytokine-cytokine receptor interaction, neuroactive ligand-receptor interaction, transcriptional misregulation in cancer, MAPK signaling pathway, etc (Figures 2E,F). These results above indicated that IS is related with the expression changes of multiple functional genes involving in a variety of biological processes.

**Table 1:** Top 30 up-regulated DEGs and 10 down-regulated DEGs in the model rats.

Genes	Description	log2FC	*p*-value	*p*-adjacent	Up/down
Ccl20	Adrenergic receptor, beta 3	10.808	0.007	0.205	Up
Cxcl2	X-linked lymphocyte-regulated 3B	8.268	0.003	0.133	Up
Ccl2	MAS-related GPR, member B2	8.127	0.000	0.000	Up
Ccl7	RIKEN cDNA 1700001O22 gene	8.113	0.000	0.000	Up
LOC108348108	Solute carrier family 7	7.881	0.000	0.011	Up
Hspa1b	Transmembrane protein 139	7.724	0.000	0.041	Up
Serpine1	Serine (or cysteine) peptidase inhibitor, clade C (antithrombin), member 1	7.216	0.001	0.060	Up
Il11	Monooxygenase, DBH-like 1	6.902	0.014	0.290	Up
Lif	Adipogenin	6.816	0.000	0.044	Up
MGC105649	Hypothetical LOC302884	6.660	0.001	0.091	Up
LOC299282	Complement factor D (adipsin)	6.499	0.009	0.238	Up
Cxcl1	Multiple EGF-like-domains 6	6.478	0.003	0.138	Up
Ccl4	C1q and tumor necrosis factor related protein 3	6.463	0.002	0.096	Up
Ccl3	Lectin, galactose binding, soluble 12	6.237	0.003	0.133	Up
Mcemp1	Cell death-inducing DFFA-like effector c	6.111	0.003	0.136	Up
Neurl3	Neuralized E3 ubiquitin protein ligase 3	6.093	0.003	0.143	Up
Dsg3	Desmoglein 3	5.723	0.007	0.195	Up
Scimp	SLP adaptor and CSK interacting membrane protein	5.637	0.003	0.141	Up
Tfpi2	Tissue factor pathway inhibitor 2	5.568	0.018	0.323	Up
Runx3	RUNX family transcription factor 3	5.566	0.002	0.108	Up
Ptx3	Pentraxin 3	5.480	0.000	0.009	Up
Il6	Interleukin 6	5.399	0.028	0.391	Up
Timp1	TIMP metallopeptidase inhibitor 1	5.349	0.005	0.168	Up
Exo1	Exonuclease 1	5.314	0.003	0.129	Up
Gsdma	Gasdermin A	5.252	0.004	0.154	Up
Il1rn	Interleukin 1 receptor antagonist	5.069	0.004	0.163	Up
AABR07060872.1	–	5.023	0.000	0.015	Up
Atf3	Activating transcription factor 3	4.973	0.001	0.078	Up
FAM187A	Family with sequence similarity 187, member A	4.851	0.000	0.000	Up
Pi15	Peptidase inhibitor 15	4.838	0.013	0.280	Up
Lhx8	LIM homeobox 8	−6.100	0.003	0.130	Down
Scn10a	Sodium voltage-gated channel alpha subunit 10	−5.240	0.015	0.295	Down
Ano2	Anoctamin 2	−5.172	0.001	0.077	Down
Brs3	Bombesin receptor subtype 3	−5.000	0.003	0.130	Down
Prlr	Prolactin receptor	−4.440	0.019	0.326	Down
Mrgprx3	MAS related GPR family member X3	−4.106	0.012	0.263	Down
AABR07031445.1	–	−4.008	0.000	0.000	Down
Tdrd9	Tudor domain containing 9	−3.832	0.026	0.380	Down
Smim35	Small integral membrane protein 35	−3.605	0.002	0.096	Down
Smyd1	SET and MYND domain containing 1	−3.476	0.009	0.240	Down
Igbp1b	Immunoglobulin (CD79A) binding protein 1b	−3.335	0.002	0.108	Down
Tacr2	Tachykinin receptor 2	−3.300	0.020	0.332	Down
Krt77	Keratin 77	−3.186	0.000	0.001	Down
Serpind1	Serpin family D member 1	−3.099	0.044	0.480	Down
Slc22a6	Solute carrier family 22 member 6	−3.063	0.016	0.303	Down

### Validation of some ischemic injury-related differentially expressed genes

**Figure 3:**
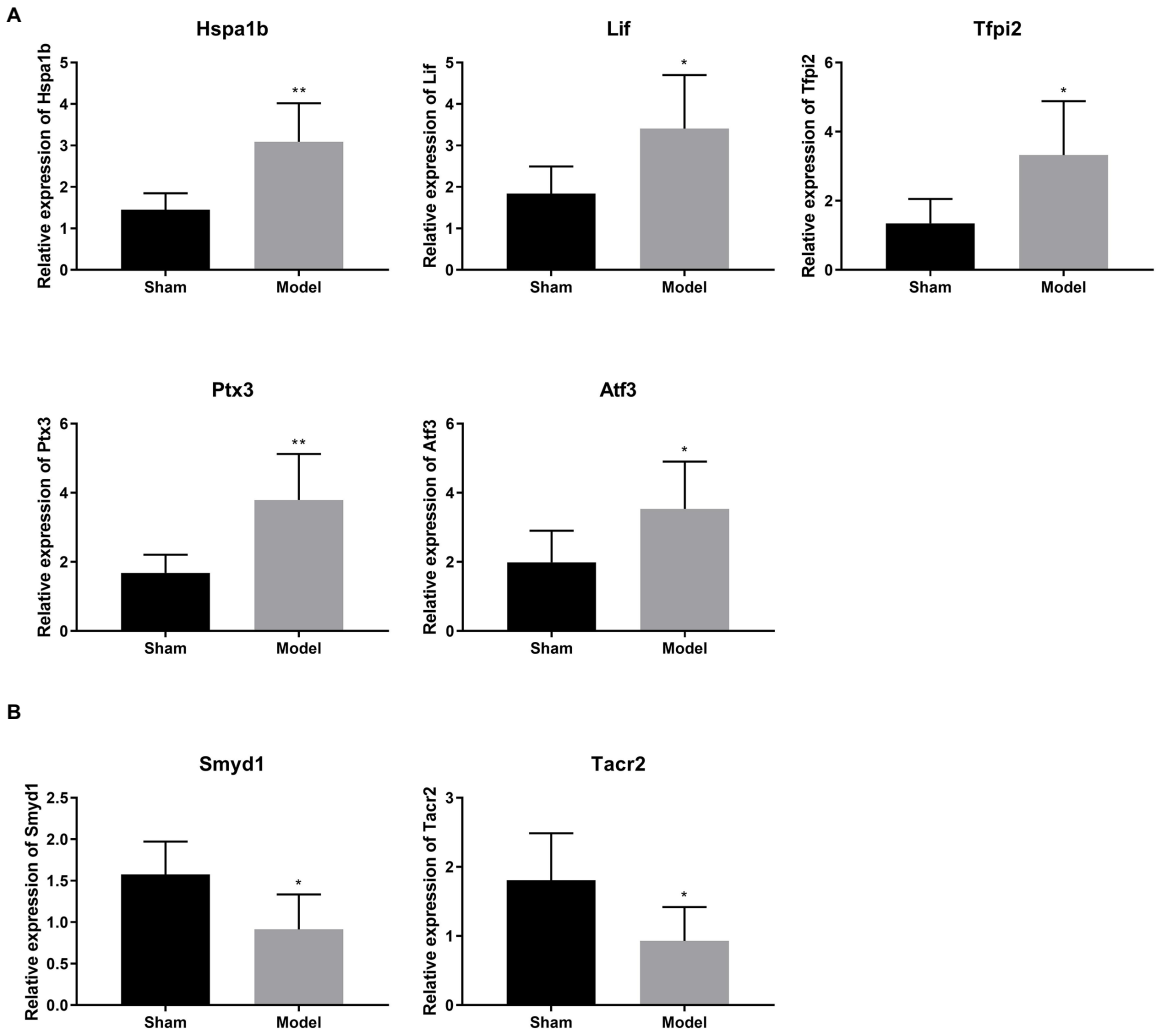
**(A,B)** Verification of 6 selected DEGs relating with ischemic injury by qRT-PCR (*N* = 6 each group). ^*^p < 0.05, ^**^p < 0.01 vs. Sham.

Several DEGs relating with ischemic injury were selected from Table 1 for validation. qRT-PCR showed that the mRNA expression of Hspa1b, Tfpi2, Ptx3, Atf3 were significantly higher in the Model group compared with those in the Sham group (*p* < 0.05; Figure 3A). On the contrary, the mRNA expression of Smyd1 and Tacr2 were significantly lower in the Model group than those in the Sham group (*p* < 0.05; Figure 3B). These results of qRT-PCR were just consistent with those of transcriptome sequencing, and indicated these DEGs might be candidates for further studies on IS.

### Knockdown of ATF3 suppresses the pathological injury of brain tissues in is rats

**Figure 4:**
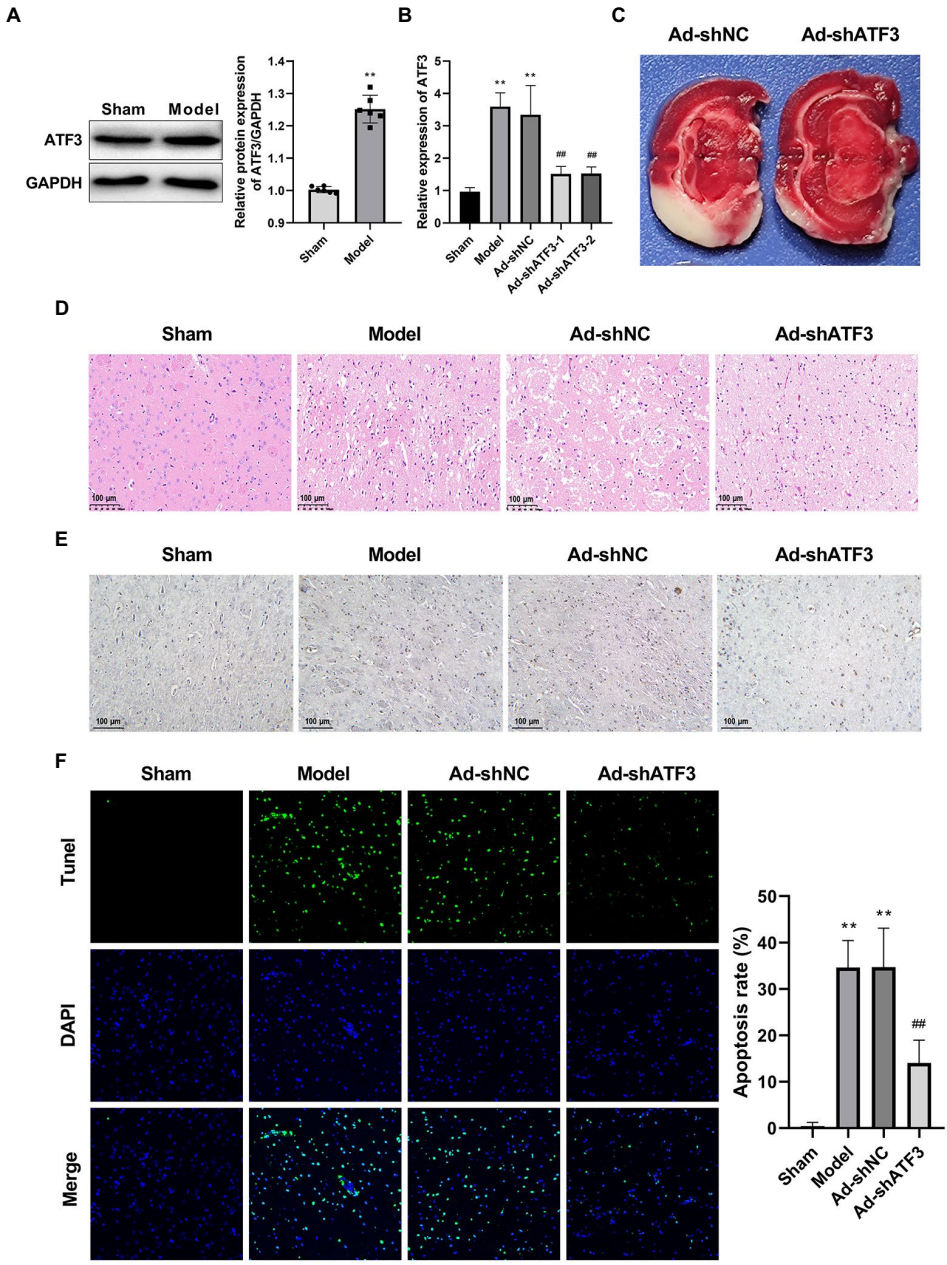
Knockdown of ATF3 suppressed the pathological injury of IS (*N* = 6 each group). **(A)** Western blot of ATF3 expression at the protein level. **(B)** qRT-PCR analysis of the knockdown efficiency of ATF3 at the mRNA level. **(C)** TTC staining. **(D)** HE staining. **(E)** IHC staining for caspase-3. **(F)** TUNEL staining. ^**^p < 0.01 vs. Sham; ^##^*p* < 0.01 vs. Model.

Among the validated DEGs, only ATF was related with both IS and ferroptosis as previously described. Therefore, we focused on ATF3 for functional studies. Western blot further identified a significantly higher protein expression of ATF3 in the Model group than that in the Sham group (*p* < 0.01; Figure 4A). Subsequently, ATF3 was knocked down in the model rats *via* injection of Ad-shATF3-1/2. As expected, the mRNA expression of ATF3 was significantly decreased by the injection of Ad-shATF3-1 and − 2 in the model rats (*p* < 0.01; Figure 4B). Interestingly, the infarcted area (white) in the model rats was obviously reduced by the injection of Ad-shATF3 (Ad-shATF3-1 was used in following assays), but not by the infection of Ad-shNC (Figure 4C). Besides, the interstitial edema, inflammatory cell infiltration, and disordered cell shape were recovered in the Ad-shATF3 group, closing to histomorphology in the Sham group (Figure 4D). Caspase-3 positive cells were also significantly reduced in the Ad-shATF3 group compared with that in the Model group (Figure 4E). The alleviated cell apoptosis in the Ad-shATF3 group was further confirmed by TUNEL assay (*p* < 0.01; Figure 4F).

### Knockdown of ATF3 inhibits the ferroptosis in brain tissues of is rats

**Figure 5:**
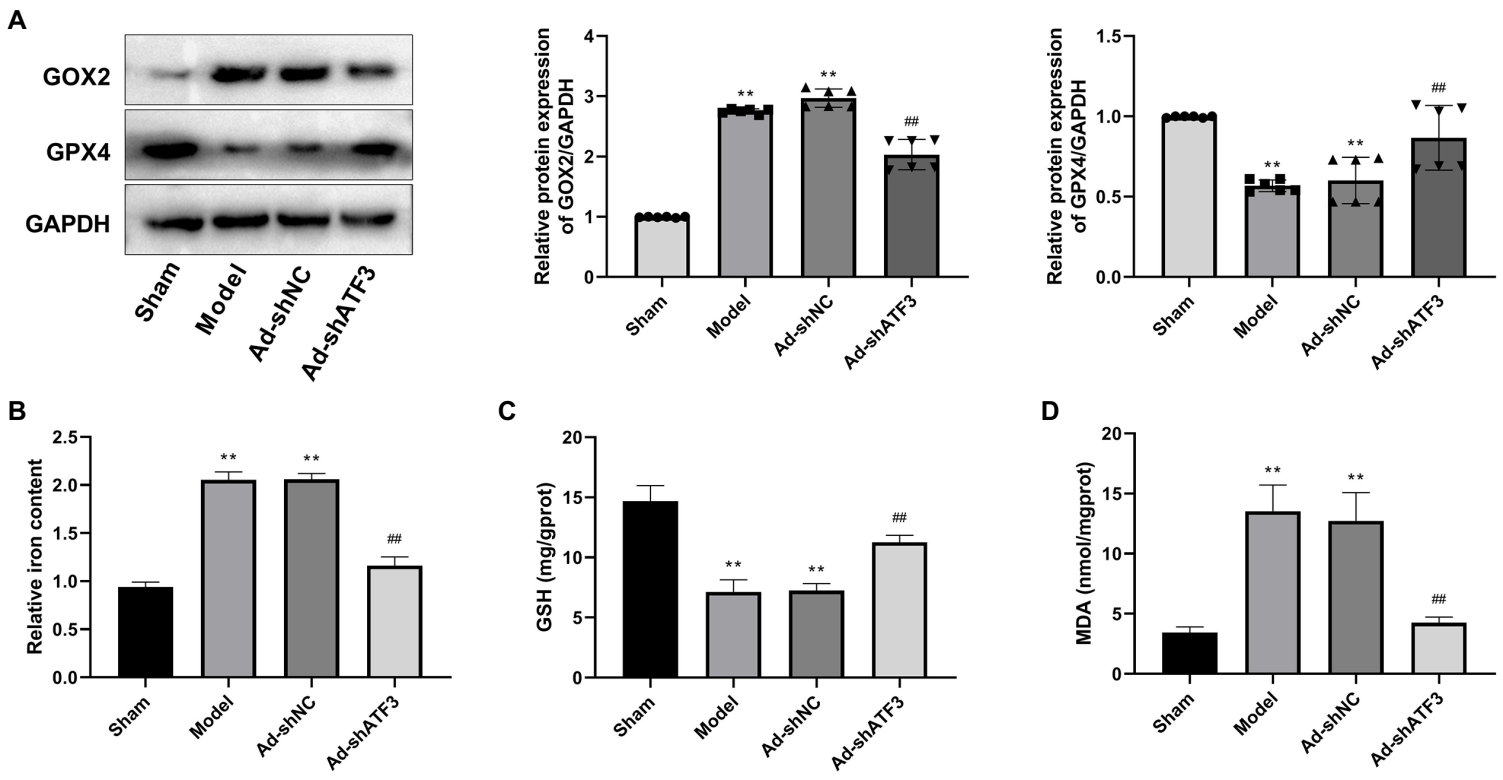
Knockdown of ATF3 inhibited ferroptosis in IS rats (*N* = 6 each group). **(A)** Western blot analysis of GOX2 and GPX4 expression at the protein level. **(B)** Iron content. **(C)** GSH content. **(D)** MDA content. ^**^p < 0.01 vs. Sham; ^##^*p* < 0.01 vs. Model.

Whether the action mechanism of ATF3 in IS is associated with the ferroptosis was further analyzed. COX2 (a negative regulator of ferroptosis) and GPX4 (a central positive regulator of ferroptosis) are two indicators of ferroptosis. The Model group showed higher protein expression of COX2 and lower protein expression of GPX4 compared to the Sham group (*p* < 0.01). The intervention of Ad-shATF3 significantly down-regulated COX2 and up-regulated GPX4 in the model rats (*p* < 0.01; Figure 5A). Excess intracellular iron is another indicator for ferroptosis, which can elevate reactive oxygen species levels and subsequent lipid peroxidation. The Model group showed a higher iron content than the Sham group (*p* < 0.01), and the abnormal increased iron content in the model rats was alleviated by the intervention of Ad-shATF3 (*p* < 0.01; Figure 5B). Furthermore, a lower content of GSH was observed in the Model group than that in the Sham group (*p* < 0.01). After knockdown of ATF3 in the model rats (the Ad-shATF3 group), the low GSH level was increased to some degrees (*p* < 0.01; Figure 5C). In contrast to GSH, the MDA level was higher in the Model group compared to the Sham group (*p* < 0.01). The intervention of Ad-shATF3 weakened the elevation of MDA level in the model rats (*p* < 0.01; Figure 5D). All the results above reflected that knockdown of ATF3 inhibited the ferroptosis in the model rats, contributing to the remission of IS.

## Discussion

IS is an major type of stroke, which is accompanied with a high risk of cognitive deficits and mortality worldwide due to ischemic neuronal damage (Zhao et al., 2022). The emerging knowledge on the molecular mechanisms involving IS facilitates the development of potential therapeutic agents (Khoshnam et al., 2017). In this study, a rat model of IS was established to discover more candidate genes with therapeutic potential against IS. After MCAO, the model rats exhibited a series of pathological changes in brain tissues, mainly including the appearance of infarction area, interstitial edema and inflammatory cell infiltration, and enhancement of cell apoptosis. These characteristics are similar to the clinical manifestations of IS in human, confirming the successful establishment of IS model in rats.

Transcriptional sequencing is a powerful method to determine the underlying gene regulatory networks in certain diseases. Until now, a variety of potential genes targeting IS have been revealed *via* transcriptional sequencing (Zhai et al., 2017; Zhou et al., 2019; Xie et al., 2020; Chen et al., 2021; Liu C. et al., 2022). In this study, transcriptional sequencing determined 699 up-regulated DEGs and 461 down-regulated DEGs in the model rats. Among the top 30 up-regulated and 10 down-regulated DEGs, 6 candidate genes (4 up-regulation and 2 down-regulation) relating with ischemic injury were selected for verification. In consistent with the sequencing results, significantly higher expression of Hspa1b, Tfpi2, and Ptx3, and lower expression of Smyd1 and Tacr2 were determined in the Model group than those in the Sham group by qRT-PCR. PTX3 is an important regulator in immune system, which is positively associated with the severity and mortality of IS (Ryu et al., 2012; Kouchaki et al., 2017). A previous study has determined that PTX3 is also a critical effector of edema resolution and glial scar formation following ischemic brain injury (Rodriguez-Grande et al., 2014). These findings indicate that PTX3 exerts a key regulatory role in the pathogenesis of IS. Although there is no direct evidence on the correlations between HSPA1B/TFPI2/SMYD1/TACR2 and IS, these verified DEGs are related with ischemic injury. For example, HSPA1B is a subtype of heat shock protein 70 that up-regulated in mice with global ischemia (James et al., 2012) and in mice with acute myocardial infarction (Liu et al., 2021). TFPI2 is a Kunitz-type serine protease inhibitor that up-regulated in young rats subjected to ischemic preconditioning (Liu et al., 2009). The up-regulation of SMYD1, a lysine methyltransferase is associated with the protective effect of exercise on cardiac function following myocardial infarction (initiated by myocardial ischemia; Liang et al., 2020). Ischemic insult alters TACR2 reactivity in the bladder of a rabbit model (Azadzoi et al., 2008). Therefore, these genes (HSPA1B, TFPI2, SMYD1, and TACR2) may also be involved in the progression of IS *via* responding to ischemia. Inhibiting of HSPA1B and TFPI2, and restoring of SMYD1 and TACR2 may be beneficial for the remission of ischemic injury. However, the detail functions of these genes in IS are rarely known. Further researches on the therapeutic potential of these genes are urgently needed.

In addition to the above DEGs, ATF3 is also a hubgene in IS. As a stress-inducible gene, ATF3 is reported to be up-regulated in peripheral blood mononuclear cells of patients with IS (Zhai et al., 2017; Li S. et al., 2022). Consistently, significantly higher expression of ATF3 was revealed in the model rats at both the mRNA and protein levels in this study. Notably, ATF3 plays an important role in regulating ferroptosis, arousing our great attention. For example, Li et al. have shown that sorafenib induces ferroptosis-mediated cardiotoxicity in mice (Li Y. et al., 2022). Wang et al. have found that quercetin relieves acute kidney injury through inhibiting ATF3-mediated ferroptosis (Wang Y. et al., 2021). Liu H. et al. (2022) have revealed that ATF3 inhibits the ferroptosis of myocardial cells following ischemia/reperfusion injury. The above evidence indicates the possibility of ferroptosis involving in the action mechanisms of ATF3 in IS. As expected, knockdown of ATF3 inhibited the ferroptosis in IS rats, evidenced by the down-regulation of COX2, up-regulation of GPX4, decreasing of iron and MDA contents, as well as increasing of GSH content in brain tissues. As an iron-dependent and lipid peroxidation-driven cell death, ferroptosis is usually activated under IS (Xu et al., 2022). Evidence has determined that the blocking of ferroptosis is a therapeutic target for IS, which can relieve ischemic brain injury (Patt et al., 1990; Palmer et al., 1994; Tuo et al., 2017; Xu et al., 2022). To combine with the positive regulatory role of ATF3 on ferroptosis, we suspect that the inhibition of ATF3-mediated ferroptosis may contribute to the remission of IS. Encouragingly, knockdown of ATF3 relieved the pathological changes of IS in brain tissues, presenting reduced infarction area and cell apoptosis, and weakened interstitial edema and inflammatory cell infiltration. Therefore, we conclude that knockdown of ATF3 suppresses the progression of IS through inhibiting ferroptosis. However, knockdown of ATF3 cannot recover the IS to a normal state. The possible reasons may be that (1) ATF3 was not completely knocked out in the model rats; (2) ATF3 is not the only gene targeting ferroptosis in IS. The discovery of more ferroptosis-related candidates in IS is still needed.

In conclusion, 699 up-regulated DEGs and 461 down-regulated DEGs were screened from a rat model of IS. PTX3, HSPA1B, TFPI2, SMYD1, and TACR2 were potential regulators in IS *via* responding to ischemia. It is noteworthy that knockdown of ATF3 contributed to the remission of IS through inhibiting ferroptosis. Our findings indicate that ATF3 is a promising therapeutic target for IS.

## Data availability statement

The data presented in the study are included in the article/Supplementary material, and deposited in the NCBI Genbank repository, accession number PRJNA903298. Further inquiries can be directed to the corresponding author.

## Ethics statement

The animal study was reviewed and approved by Ethics Committee of Ganzhou People's Hospital.

## Author contributions

JY and FZ contributed to the conception, design and analysis of data, performed the data analyses, and wrote the manuscript. BL and QL contributed to the conception of the study and wrote the manuscript. GZ contributed significantly to analysis and manuscript preparation and wrote the manuscript. All authors contributed to the article and approved the submitted version.

## Funding

This study was supported by Hospital Doctoral start-up fund.

## Conflict of interest

The authors declare that the research was conducted in the absence of any commercial or financial relationships that could be construed as a potential conflict of interest.

## Publisher’s note

All claims expressed in this article are solely those of the authors and do not necessarily represent those of their affiliated organizations, or those of the publisher, the editors and the reviewers. Any product that may be evaluated in this article, or claim that may be made by its manufacturer, is not guaranteed or endorsed by the publisher.
