# Supplementary figures and images for: Knockdown of ATF3 suppresses the progression of ischemic stroke through inhibiting ferroptosis
Source: Front Mol Neurosci. 2023 Jan 18;15:1079338. doi: 10.3389/fnmol.2022.1079338 (PMC9890179; doi:10.3389/fnmol.2022.1079338)

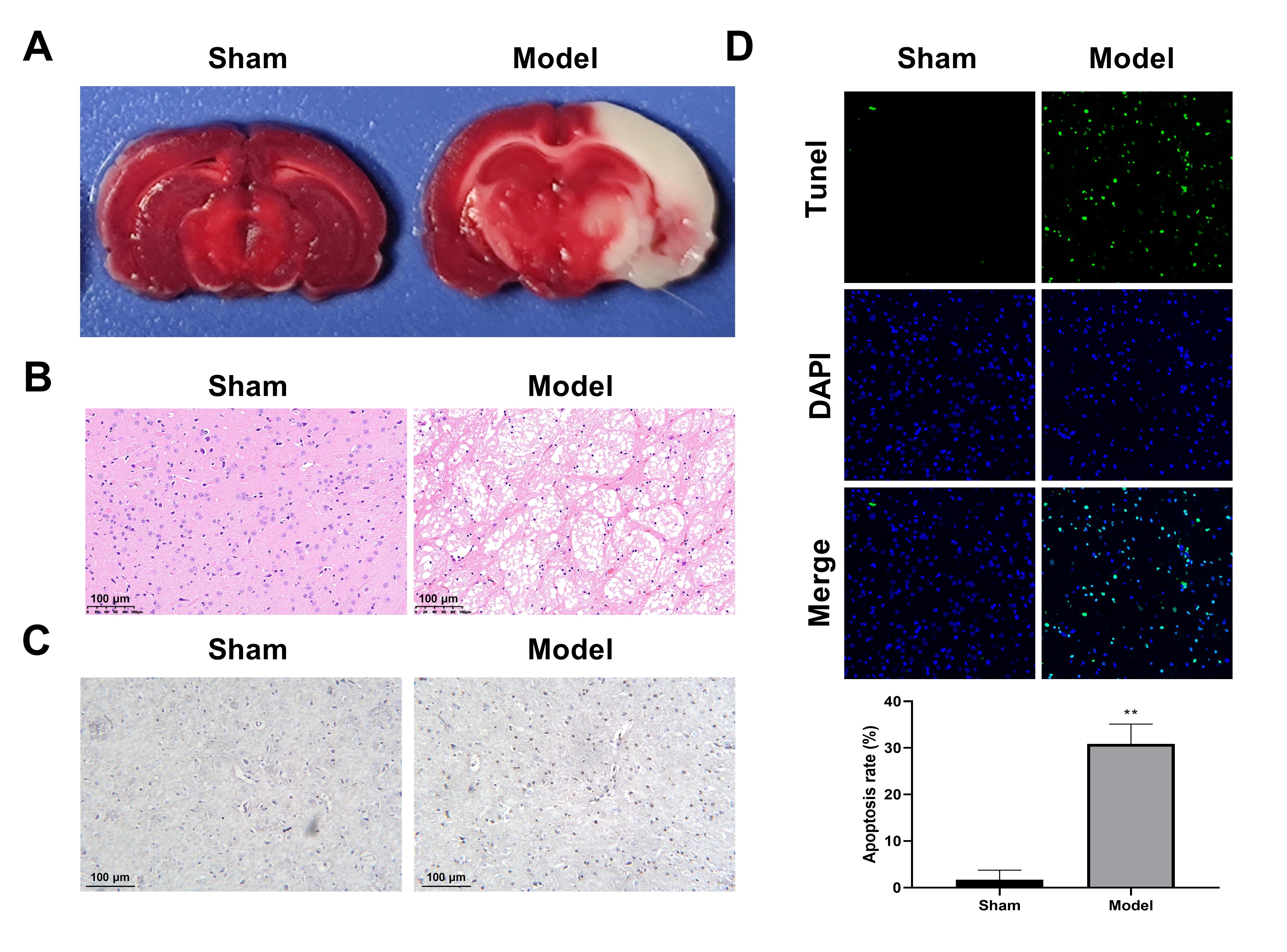

Supplement: Supplementary file 1 [file Data_Sheet_1.ZIP › Fig.1/Fig.1.jpg]

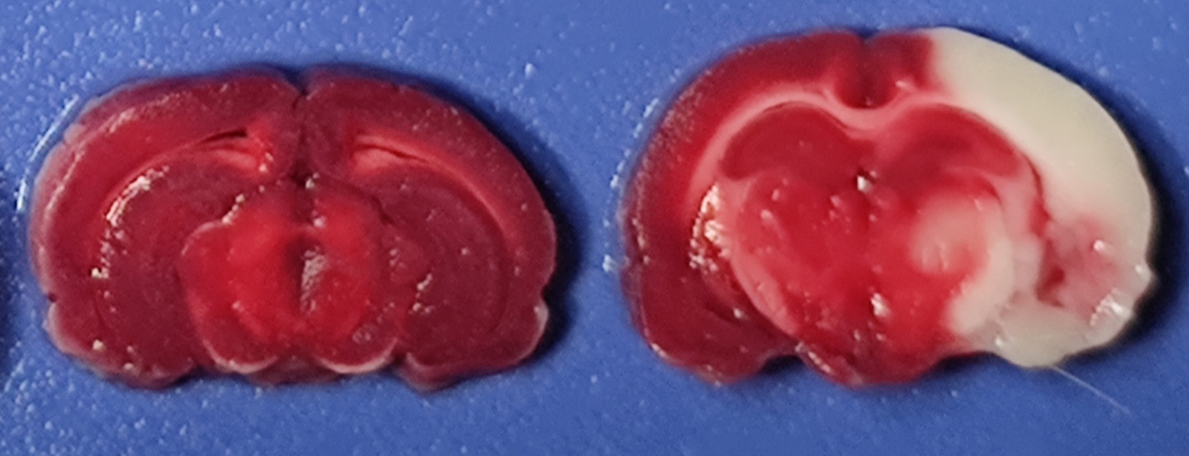

Supplement: Supplementary file 1 [file Data_Sheet_1.ZIP › Fig.1/Fig.1A-TTC staining/Fig.1A.jpg]

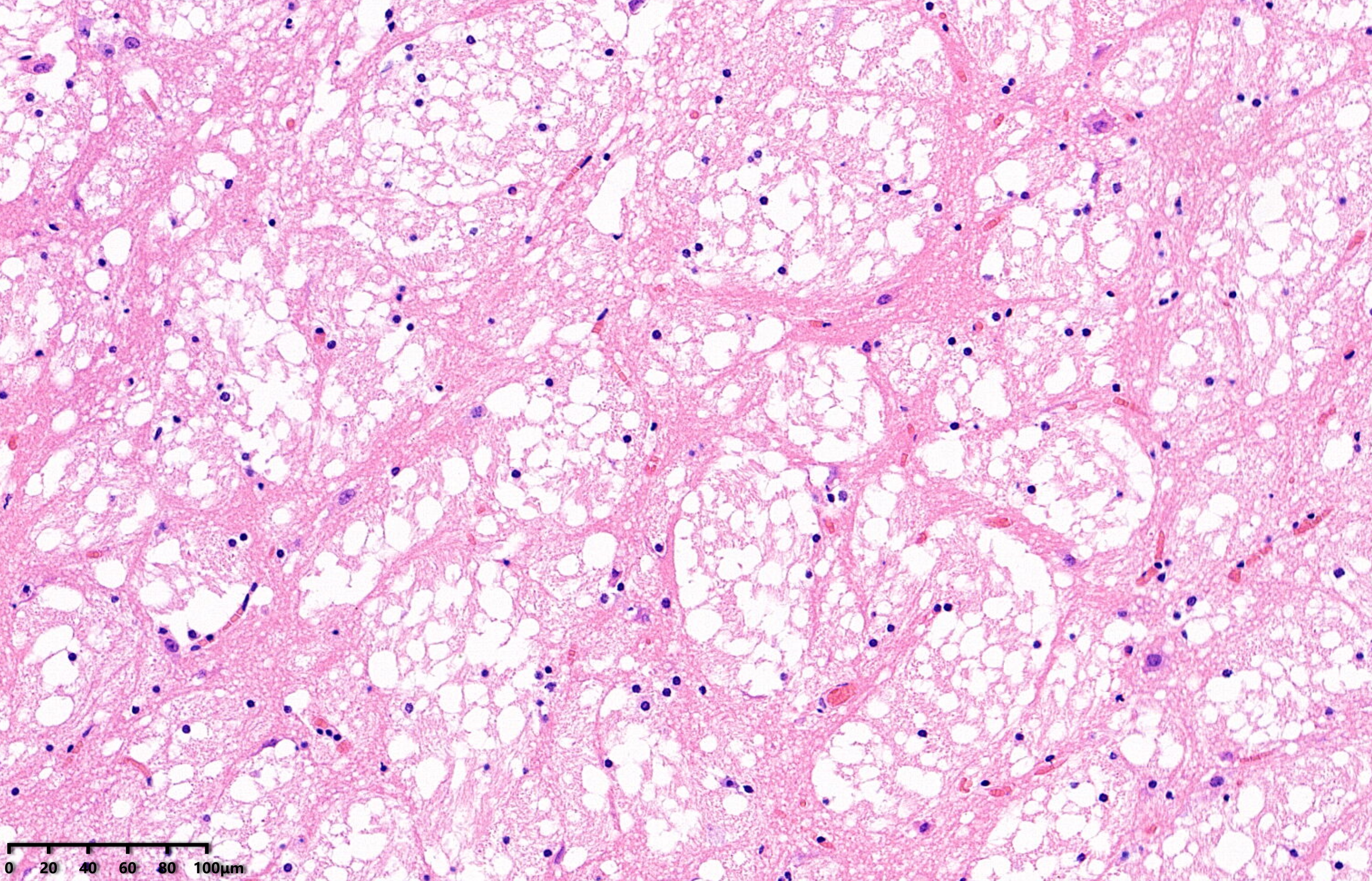

Supplement: Supplementary file 1 [file Data_Sheet_1.ZIP › Fig.1/Fig.1B-HE staining/Fig.1B-Model.jpg]

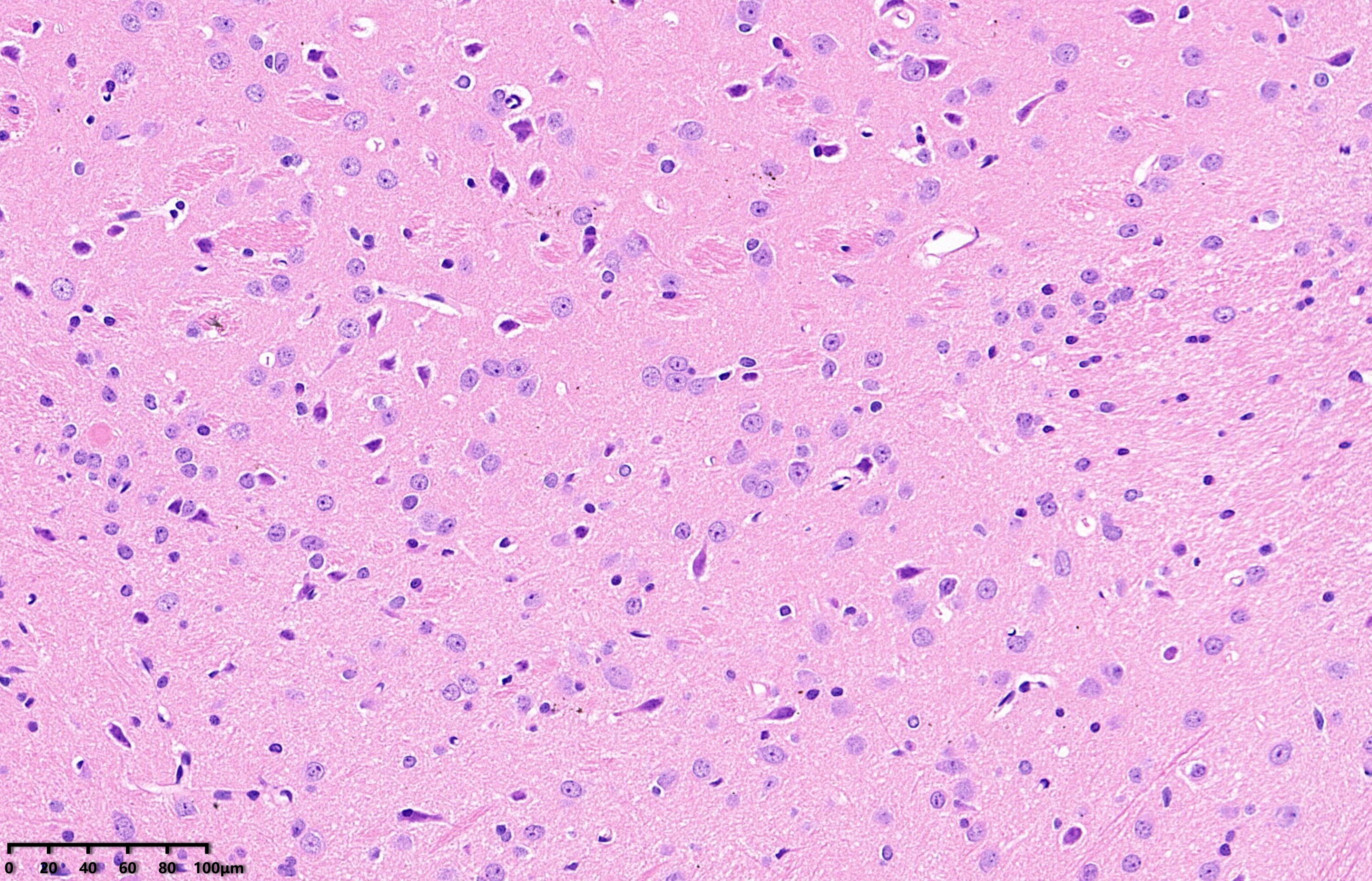

Supplement: Supplementary file 1 [file Data_Sheet_1.ZIP › Fig.1/Fig.1B-HE staining/Fig.1B-Sham.jpg]

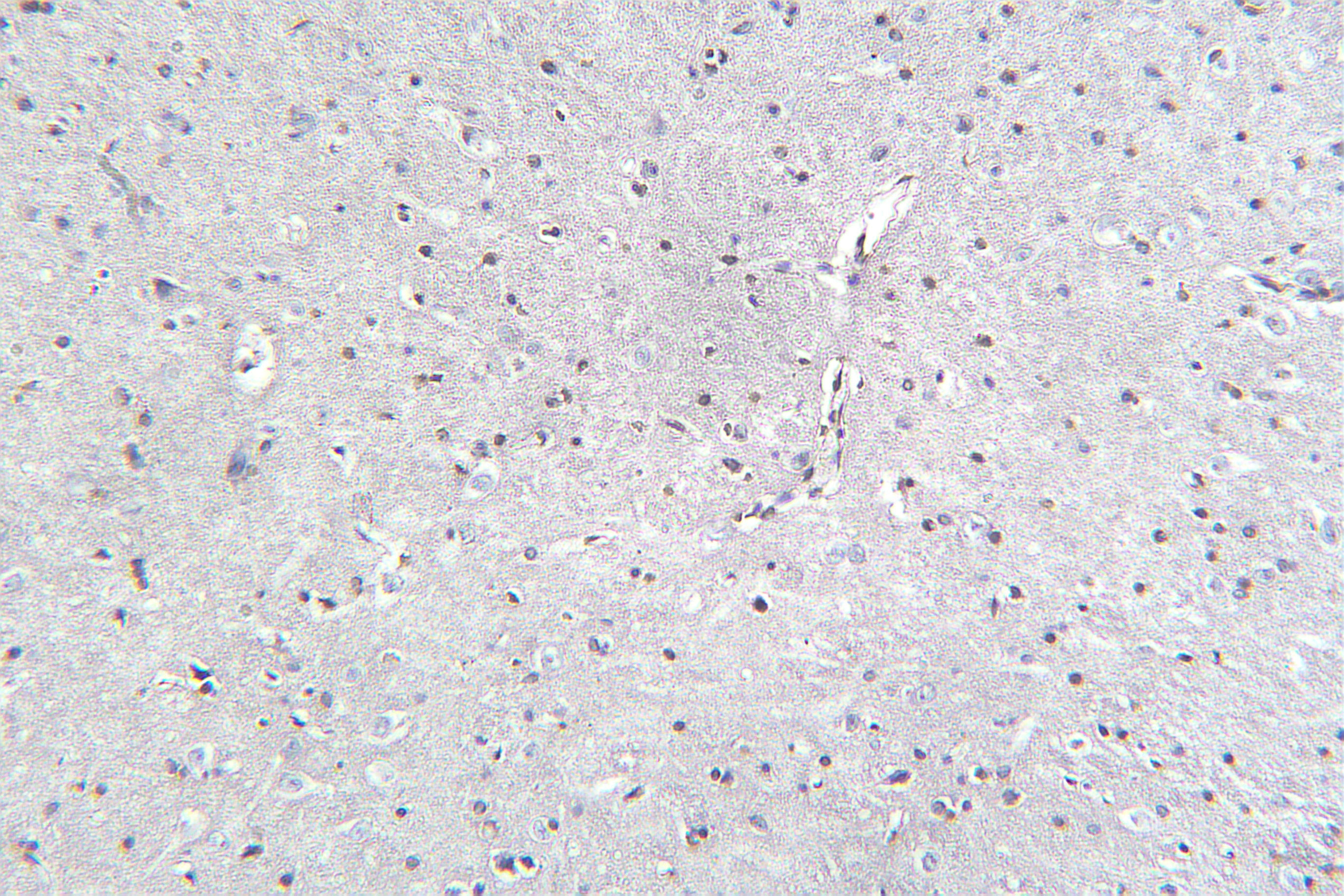

Supplement: Supplementary file 1 [file Data_Sheet_1.ZIP › Fig.1/Fig.1C-IHC staining/Fig.1C-Model.jpg]

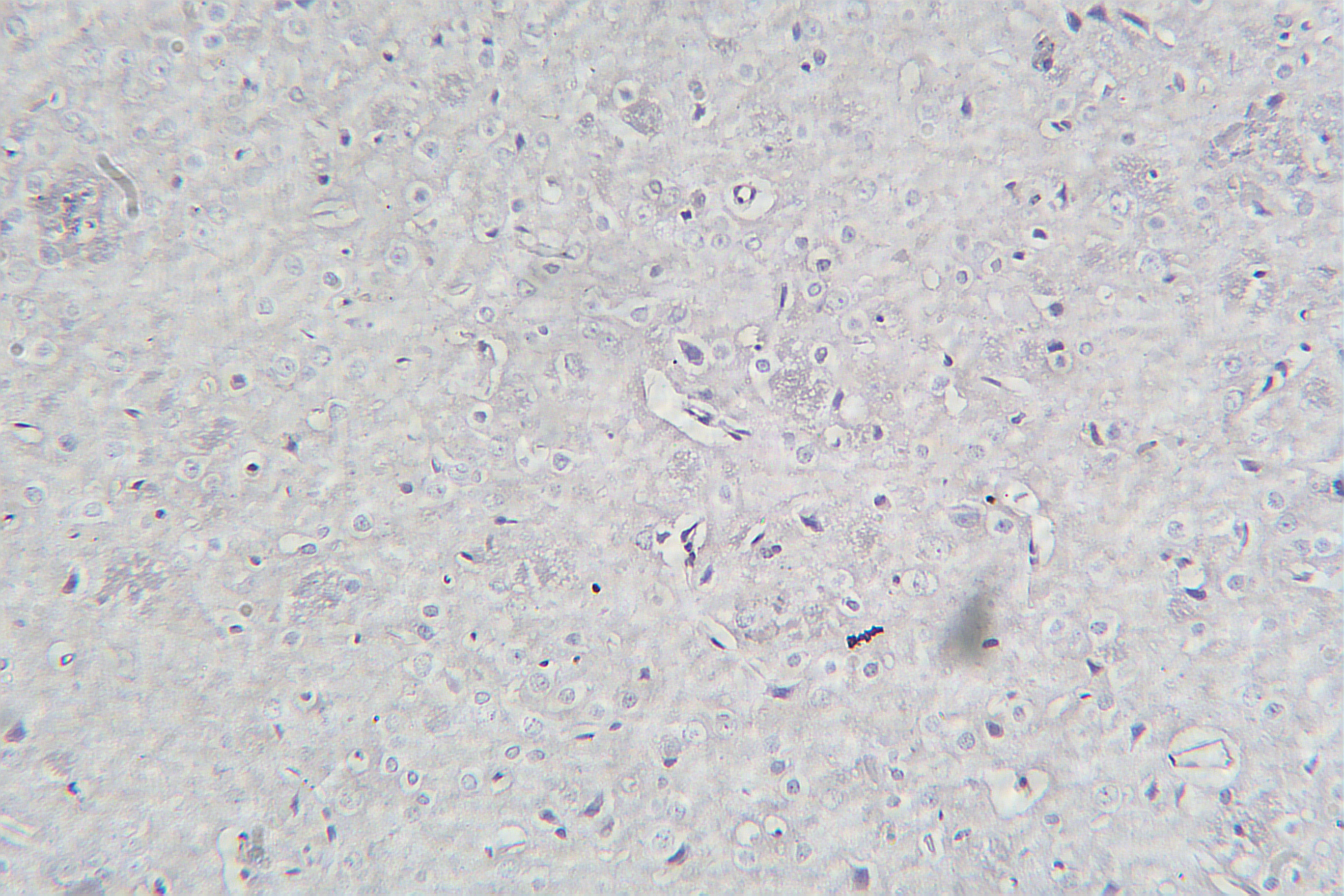

Supplement: Supplementary file 1 [file Data_Sheet_1.ZIP › Fig.1/Fig.1C-IHC staining/Fig.1C-Sham.jpg]

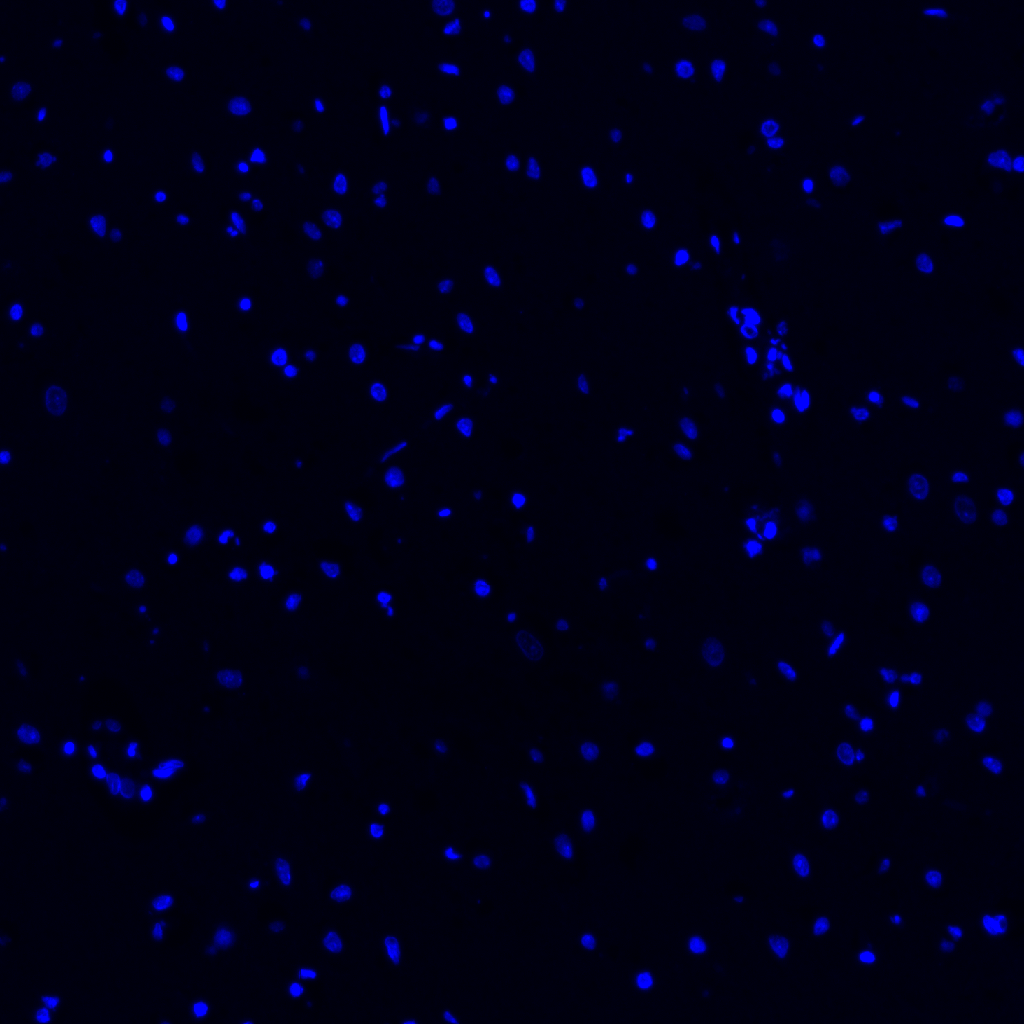

Supplement: Supplementary file 1 [file Data_Sheet_1.ZIP › Fig.1/Fig.1D-TUNEL assay/Fig.1D-Model-DAPI.tiff]

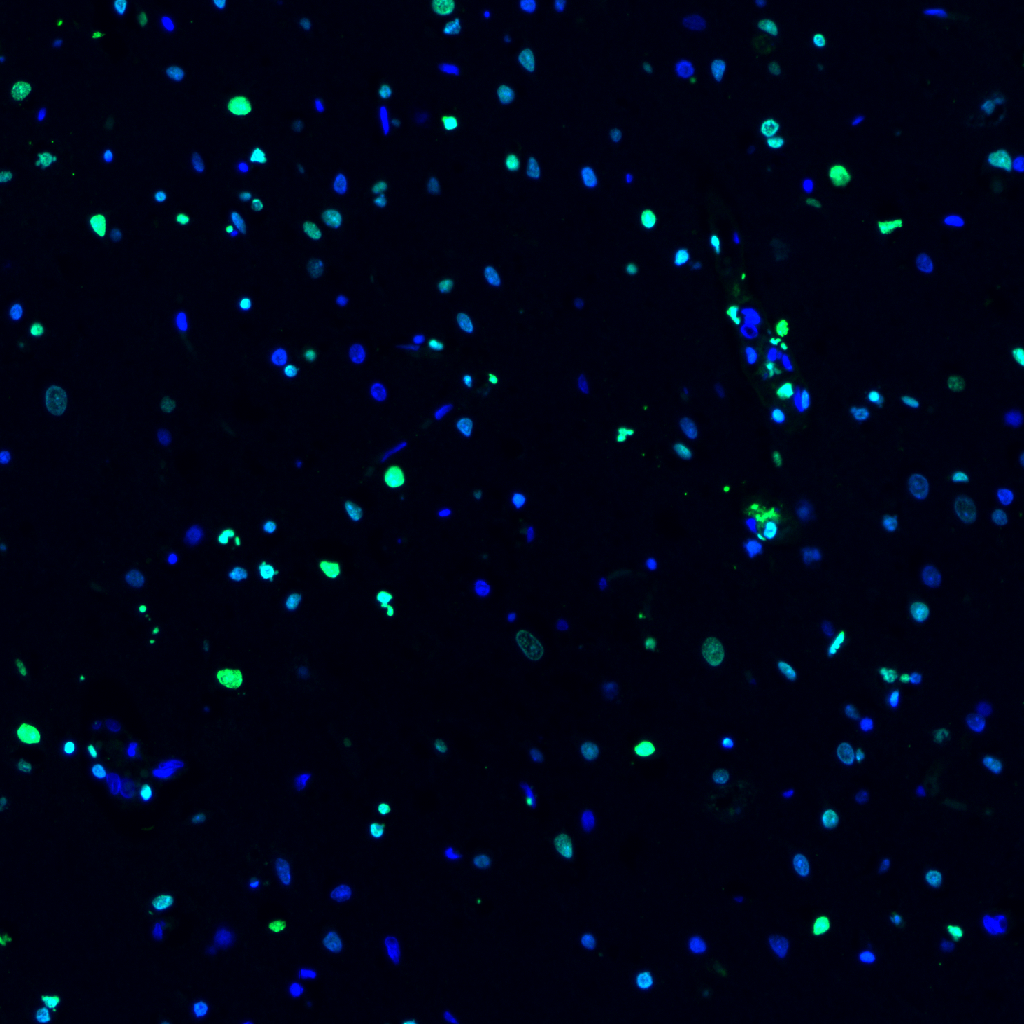

Supplement: Supplementary file 1 [file Data_Sheet_1.ZIP › Fig.1/Fig.1D-TUNEL assay/Fig.1D-Model-Merge.tiff]

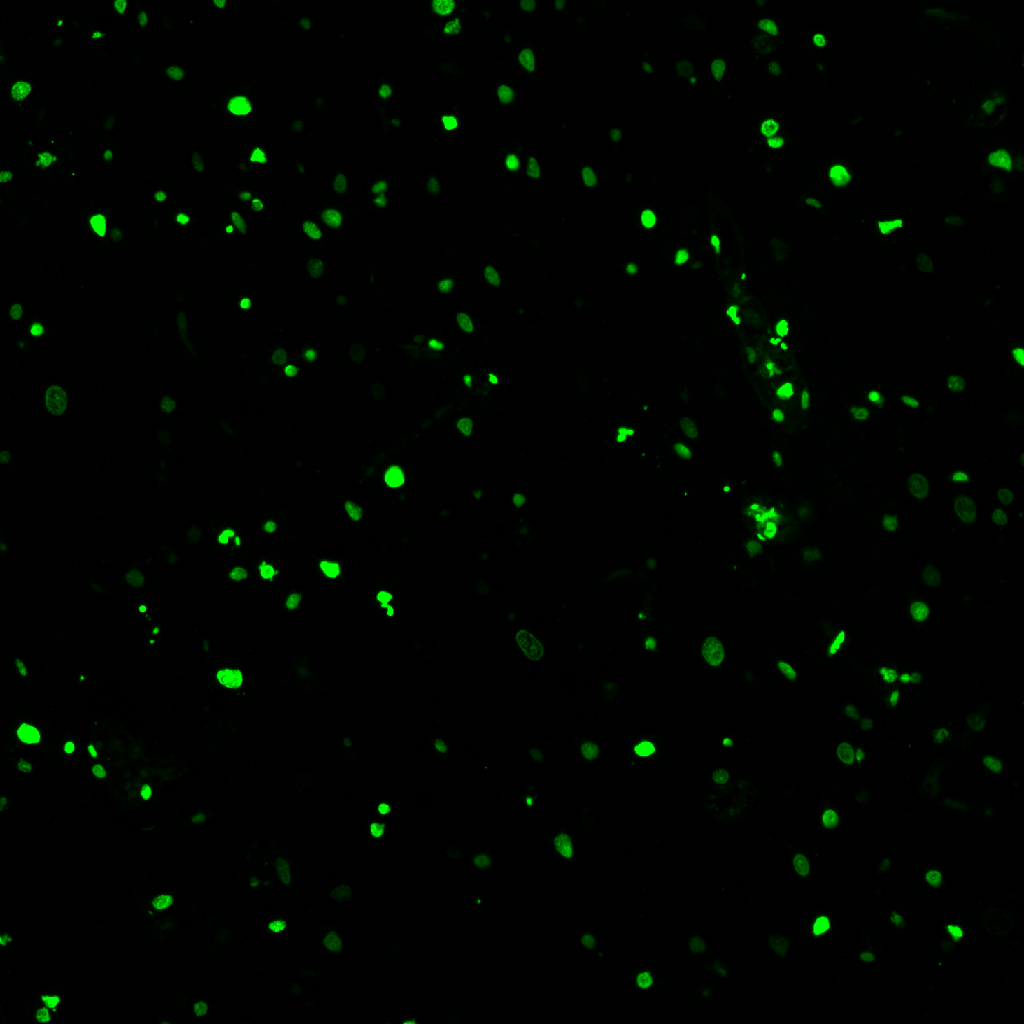

Supplement: Supplementary file 1 [file Data_Sheet_1.ZIP › Fig.1/Fig.1D-TUNEL assay/Fig.1D-Model-Tunel.tiff]

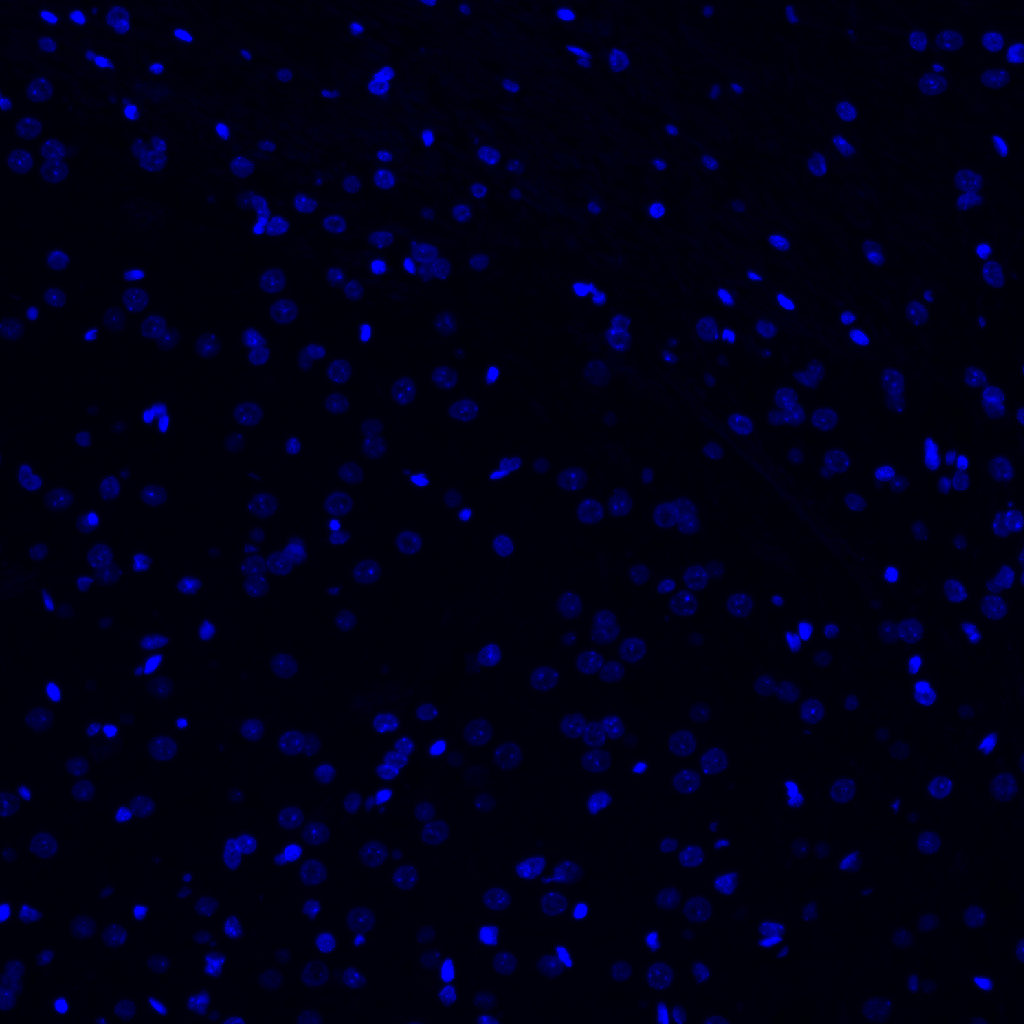

Supplement: Supplementary file 1 [file Data_Sheet_1.ZIP › Fig.1/Fig.1D-TUNEL assay/Fig.1D-Sham-DAPI.tiff]

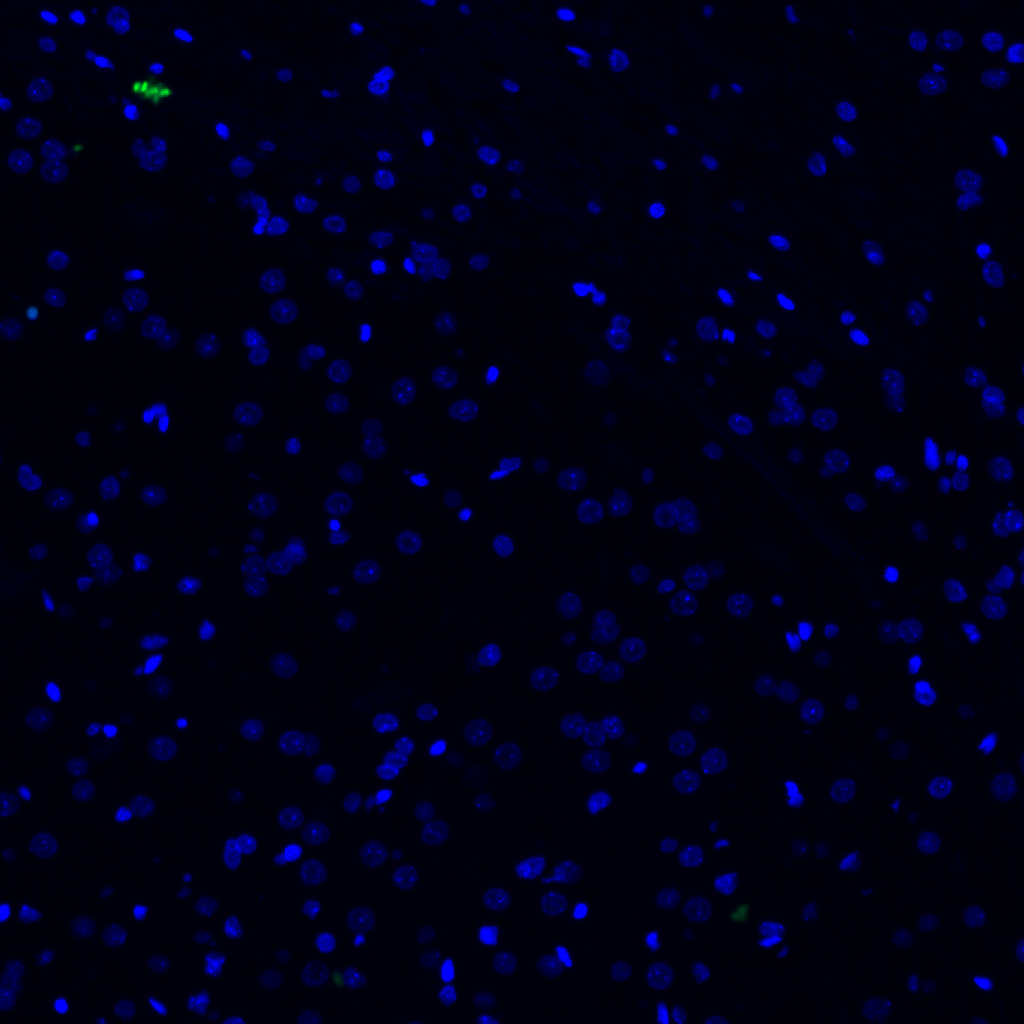

Supplement: Supplementary file 1 [file Data_Sheet_1.ZIP › Fig.1/Fig.1D-TUNEL assay/Fig.1D-Sham-Merge.tiff]

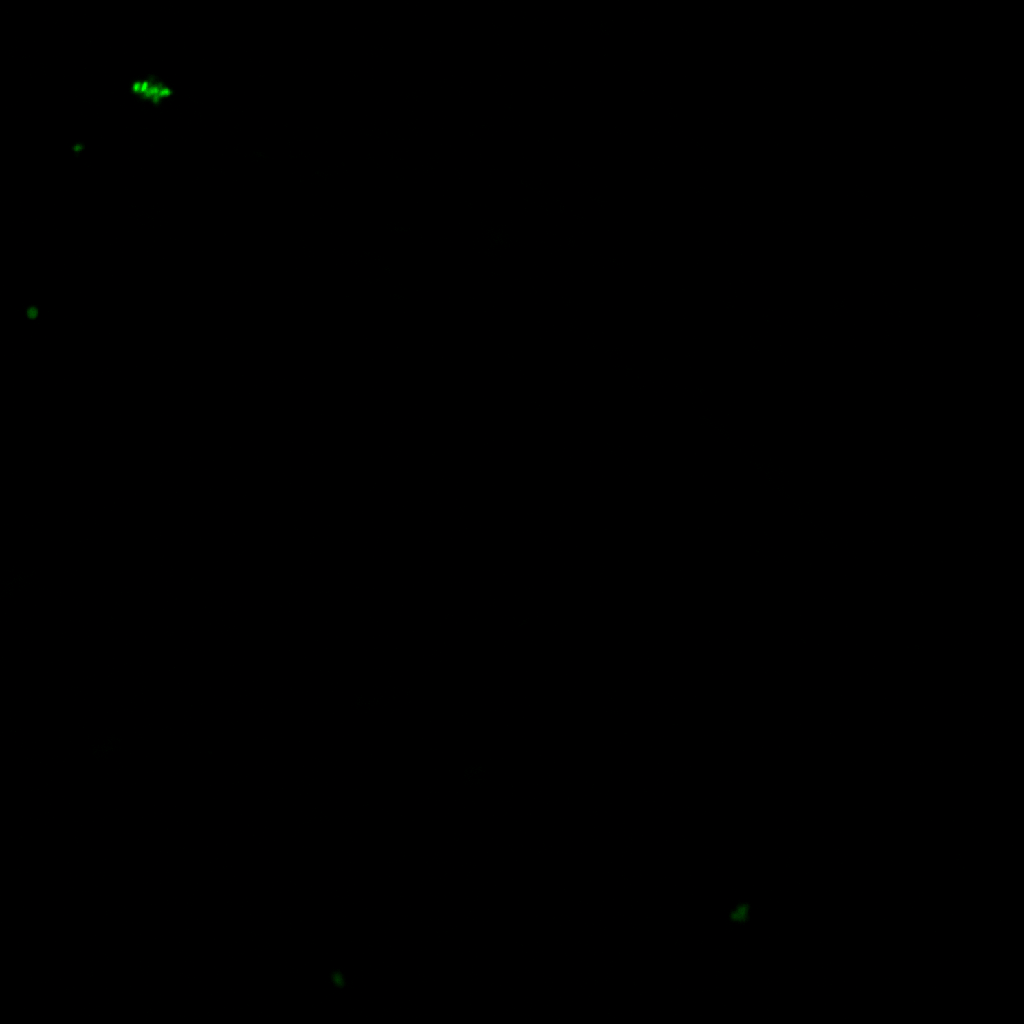

Supplement: Supplementary file 1 [file Data_Sheet_1.ZIP › Fig.1/Fig.1D-TUNEL assay/Fig.1D-Sham-Tunel.tiff]

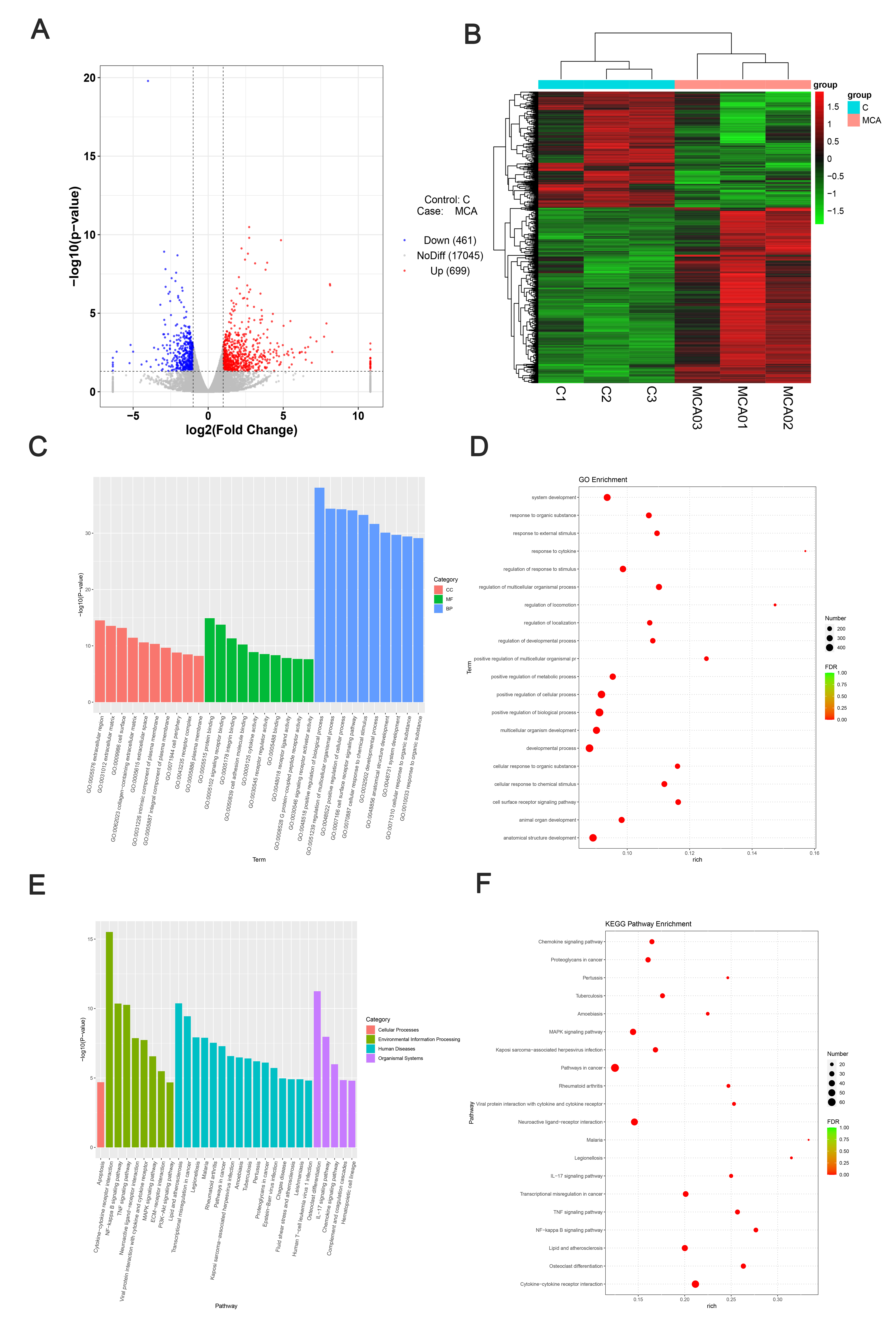

Supplement: Supplementary file 2 [file Data_Sheet_2.ZIP › Fig.2/Fig.2.jpg]

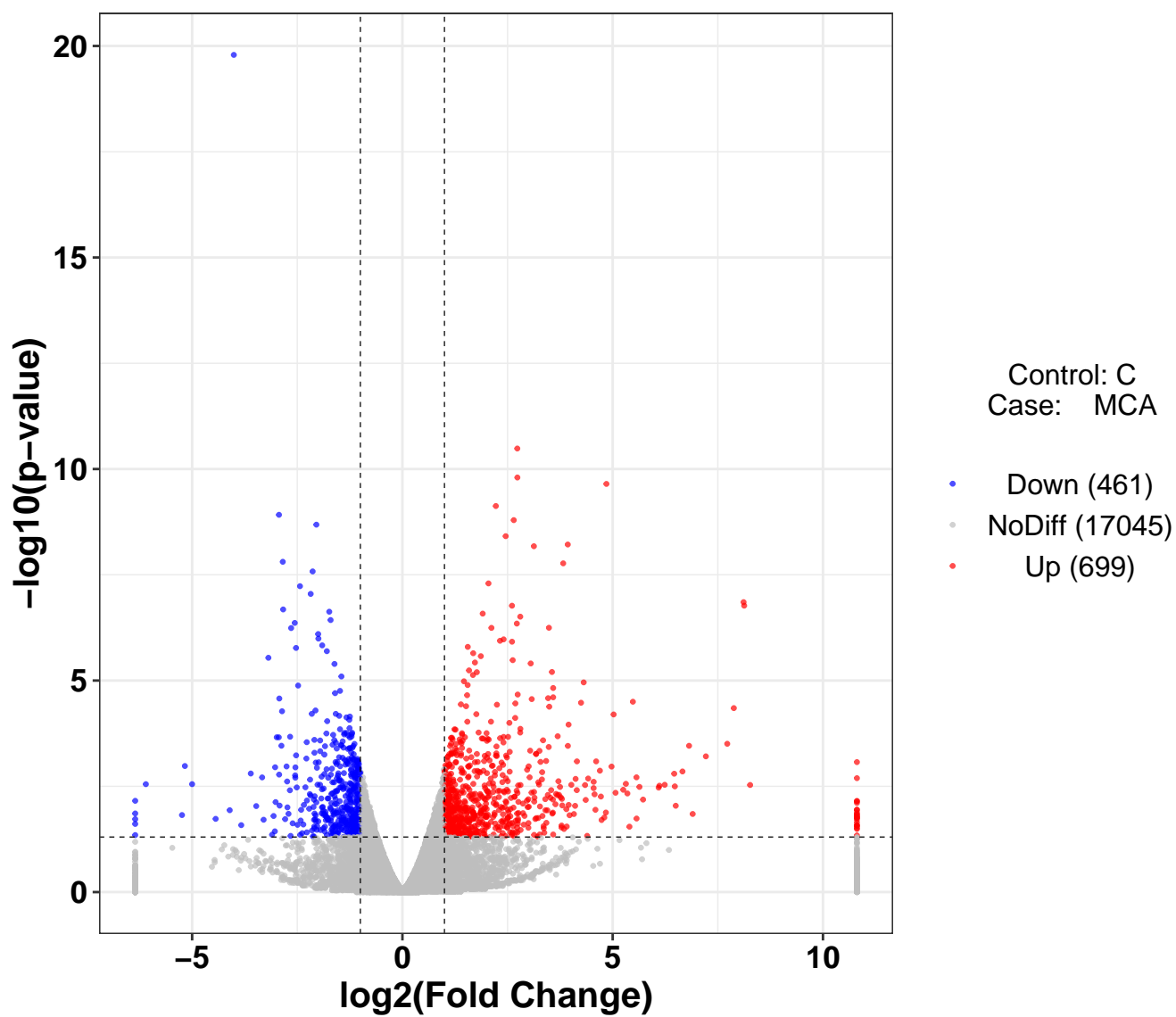

Supplement: Supplementary file 2 [file Data_Sheet_2.ZIP › Fig.2/Fig.2A/Fig.2A-Volcano plot.pdf]

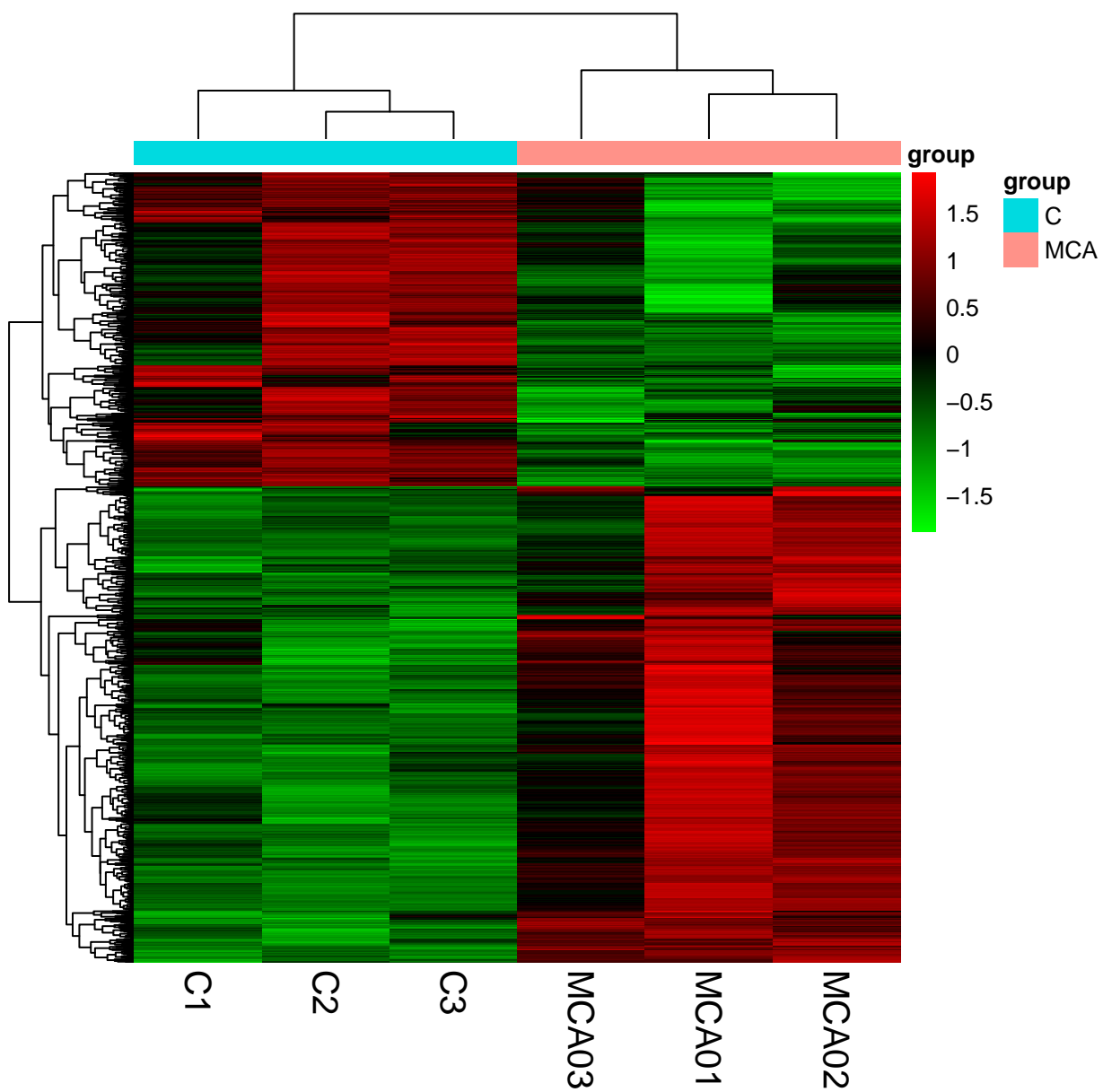

Supplement: Supplementary file 2 [file Data_Sheet_2.ZIP › Fig.2/Fig.2B/Fig.2B-heatmap.pdf]

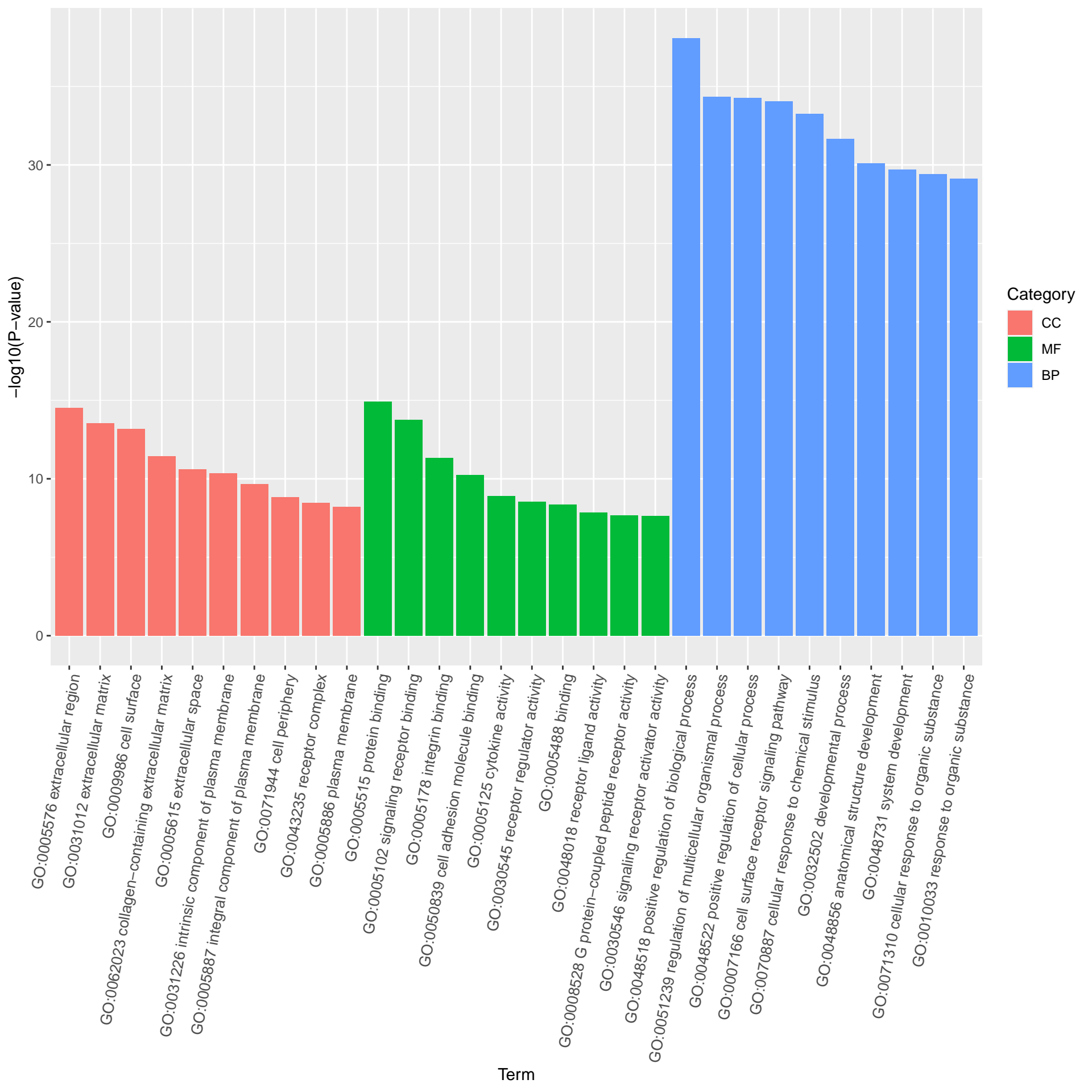

Supplement: Supplementary file 2 [file Data_Sheet_2.ZIP › Fig.2/Fig.2C-D/Fig.2C-GO-Column chart.pdf]

# GO Enrichment

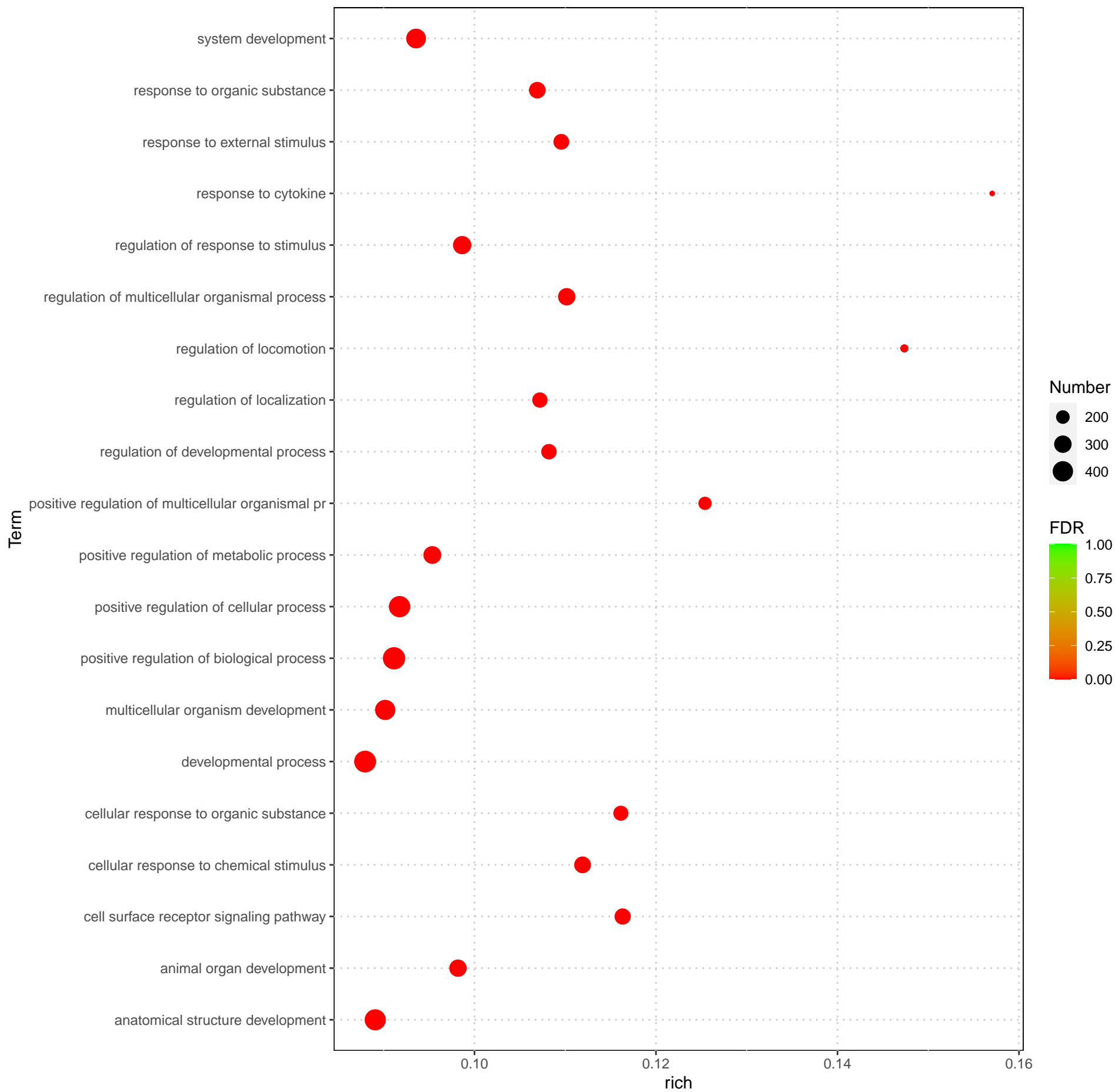

Supplement: Supplementary file 2 [file Data_Sheet_2.ZIP › Fig.2/Fig.2C-D/Fig.2D-GO-Bubble chart.pdf]

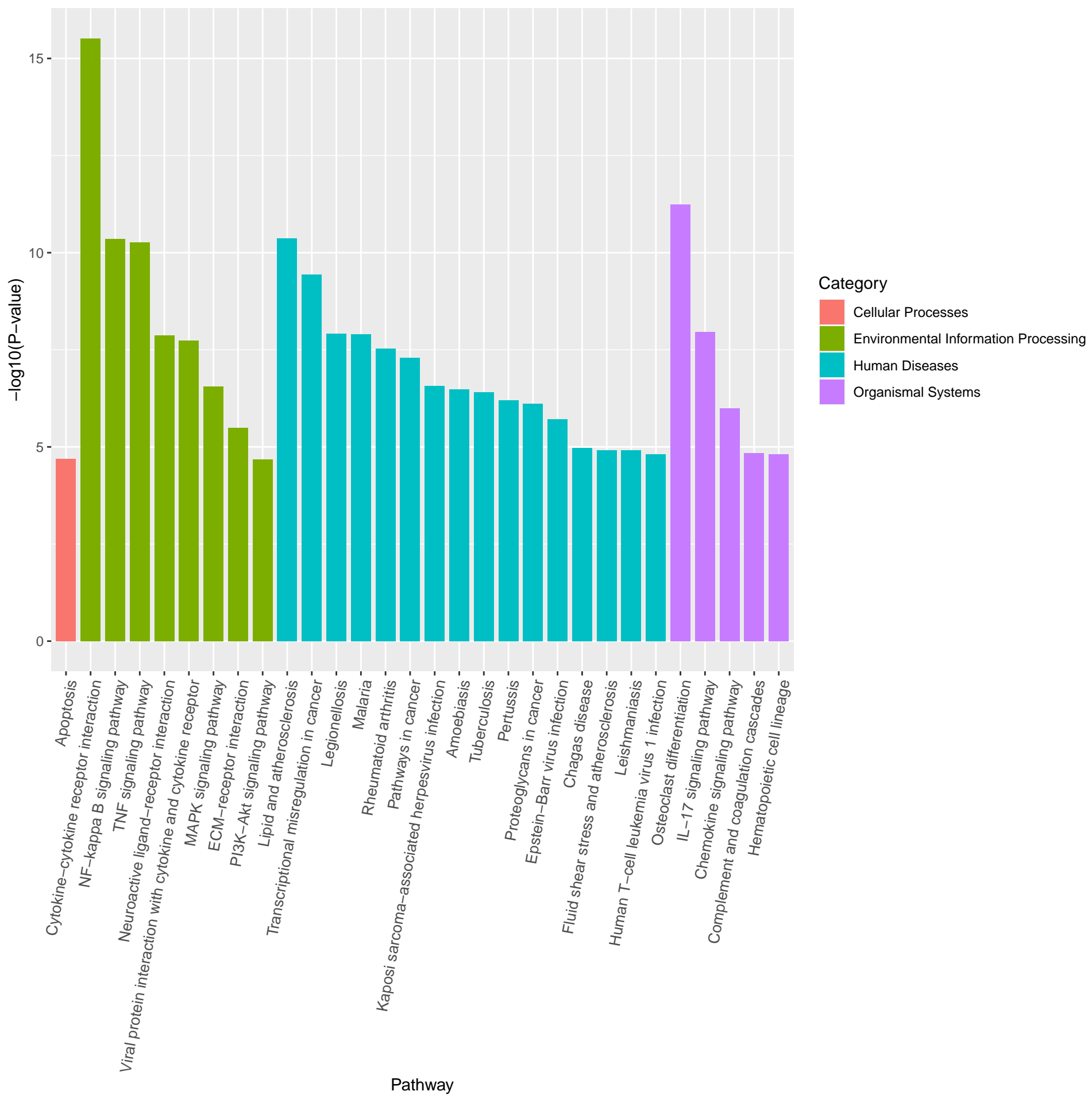

Supplement: Supplementary file 2 [file Data_Sheet_2.ZIP › Fig.2/Fig.2E-F/Fig.2E-KEGG-Column chart.pdf]

# KEGG Pathway Enrichment

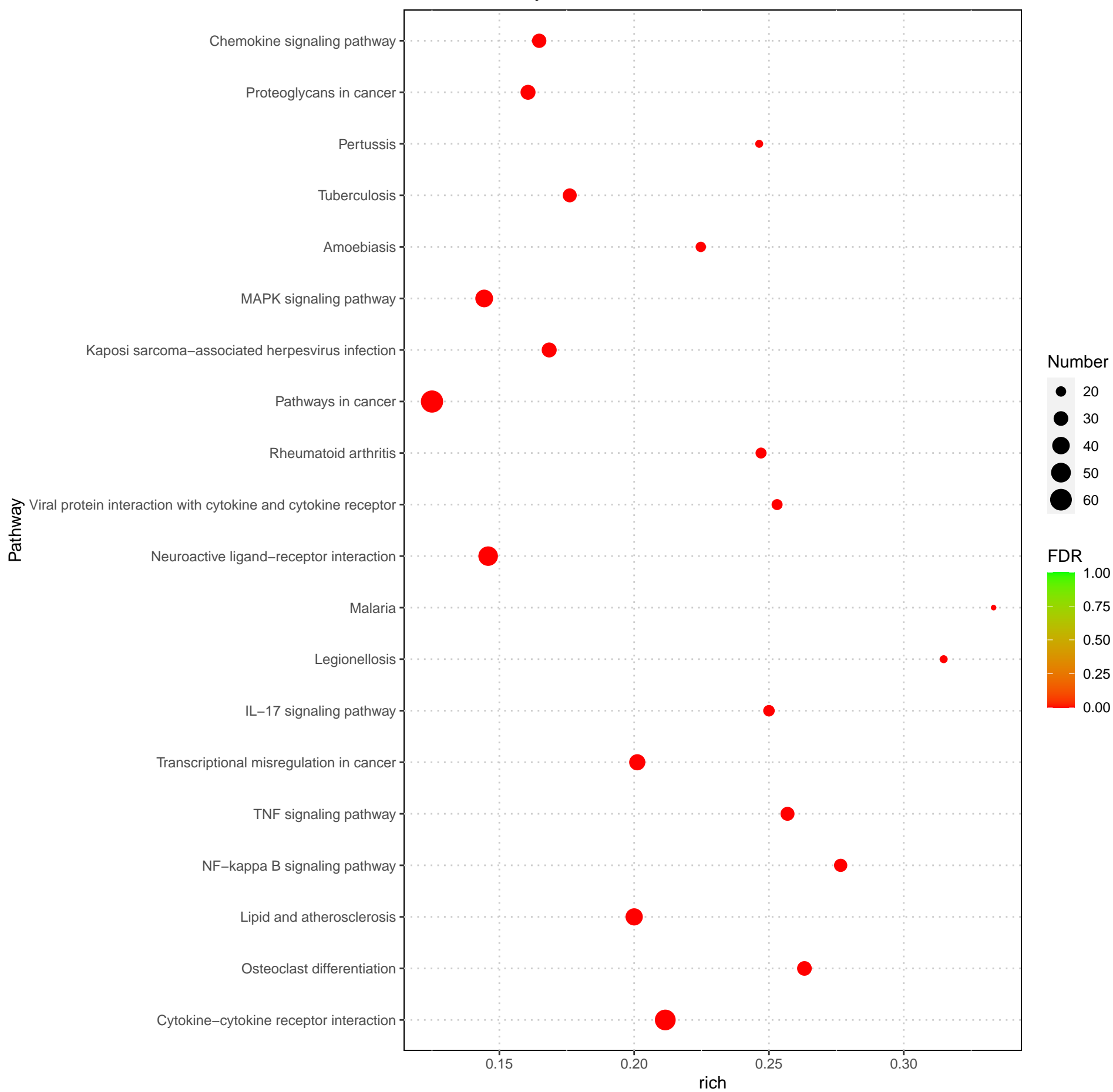

Supplement: Supplementary file 2 [file Data_Sheet_2.ZIP › Fig.2/Fig.2E-F/Fig.2F-KEGG-Bubble chart.pdf]

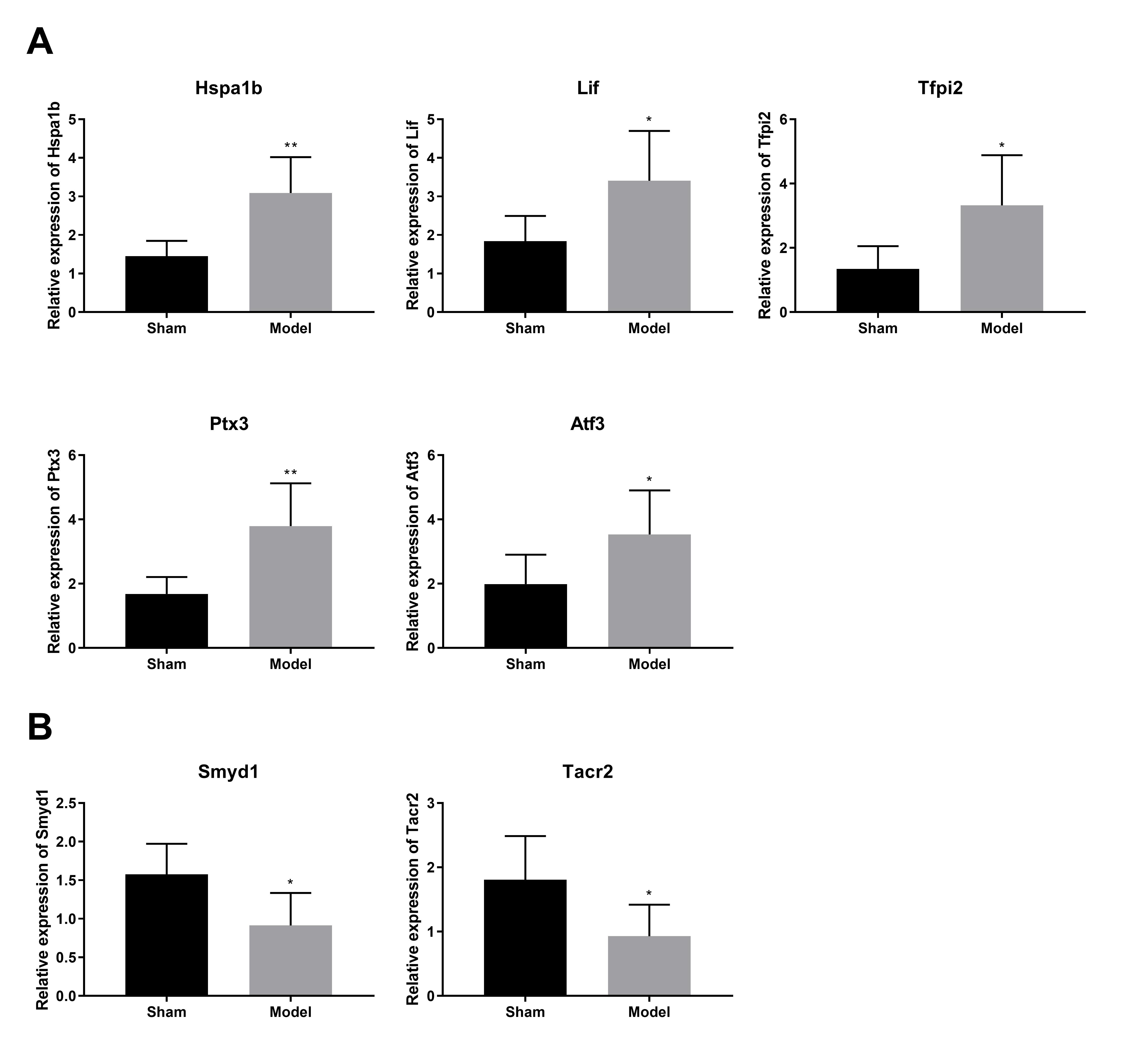

Supplement: Supplementary file 3 [file Data_Sheet_3.ZIP › Fig.3/Fig.3.jpg]

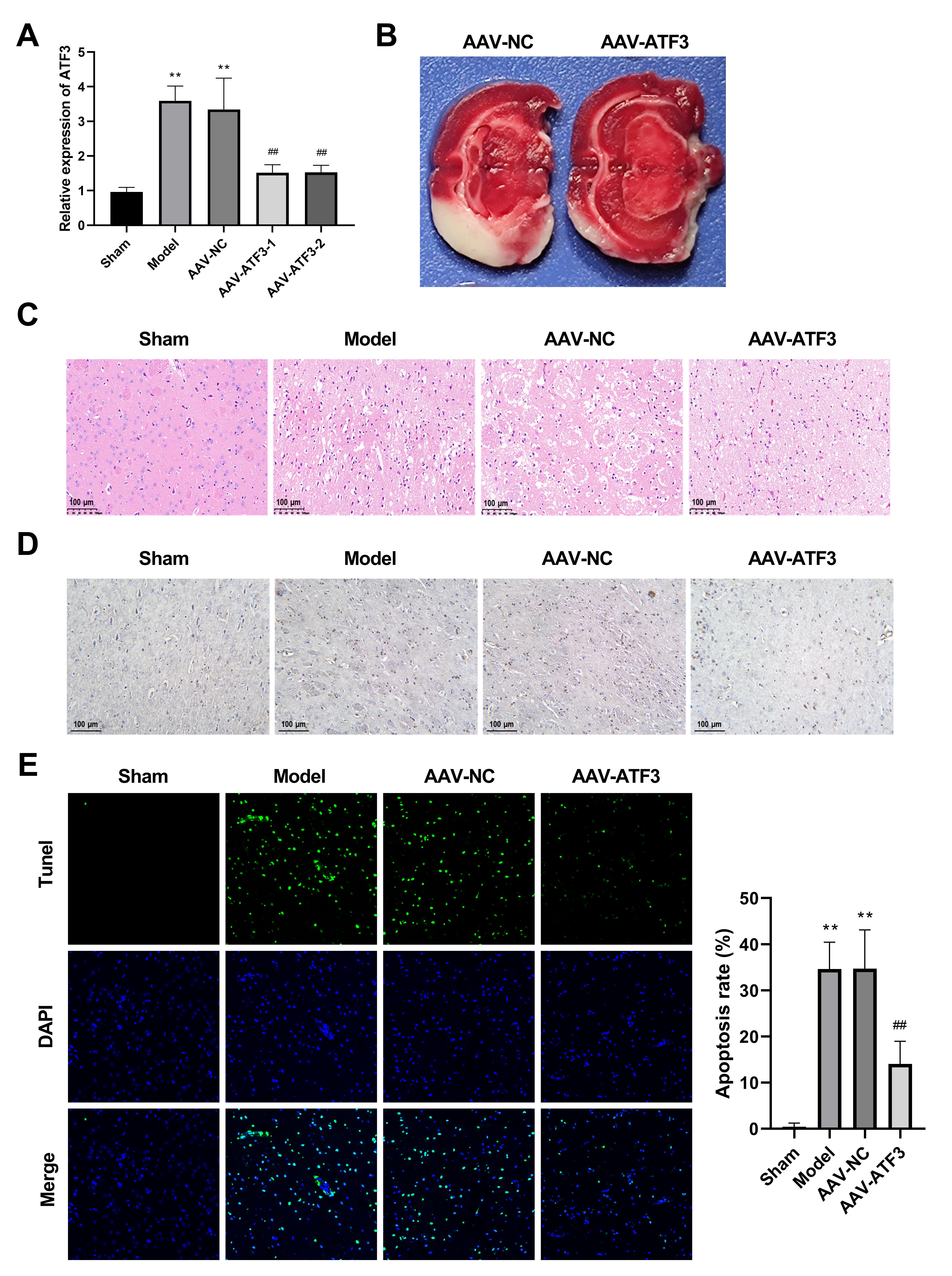

Supplement: Supplementary file 4 [file Data_Sheet_4.ZIP › Fig.4/Fig.4.jpg]

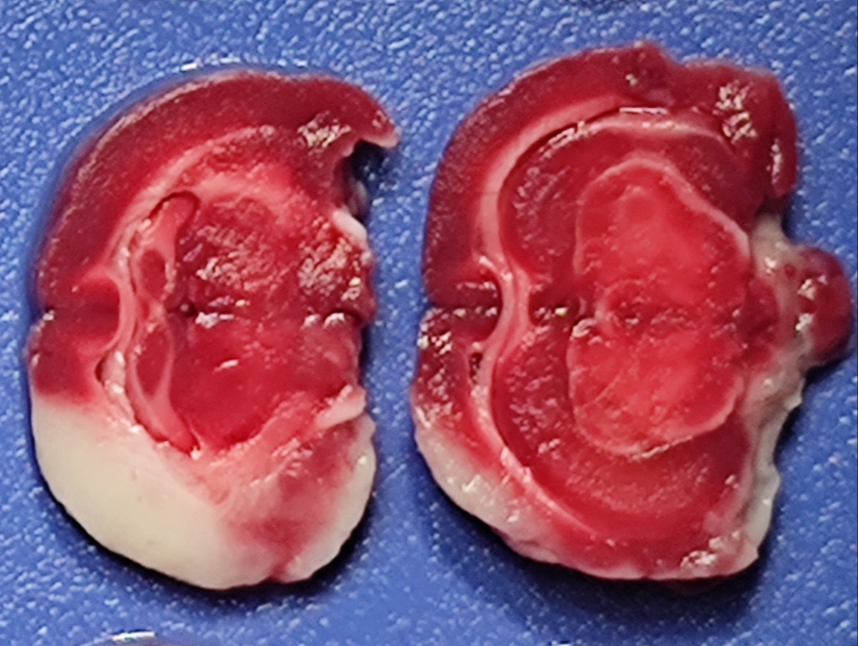

Supplement: Supplementary file 4 [file Data_Sheet_4.ZIP › Fig.4/Fig.4B-TTC staining/Fig.4B.jpg]

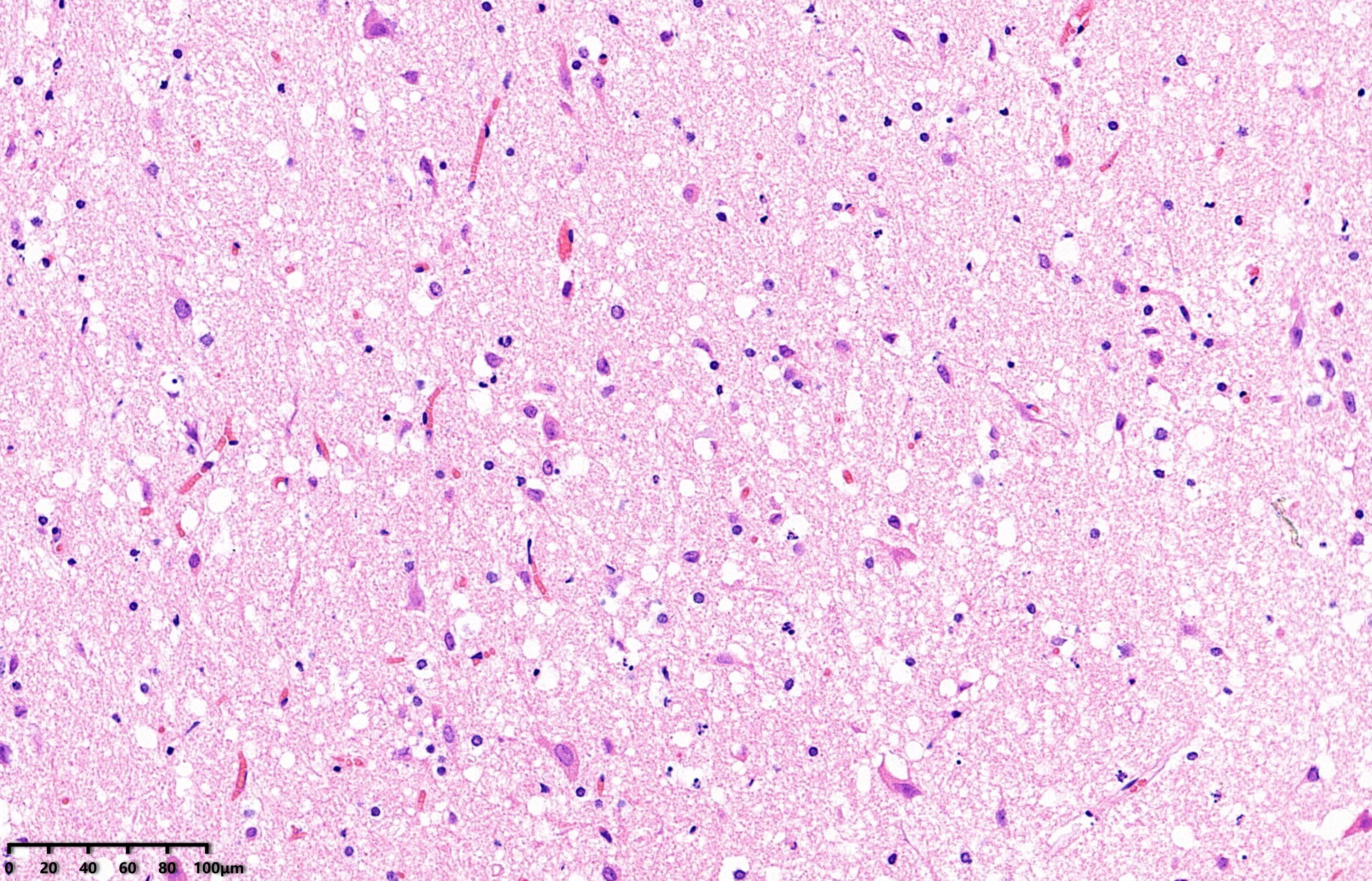

Supplement: Supplementary file 4 [file Data_Sheet_4.ZIP › Fig.4/Fig.4C-HE staining/Fig.4C-AAV-ATF3.jpg]

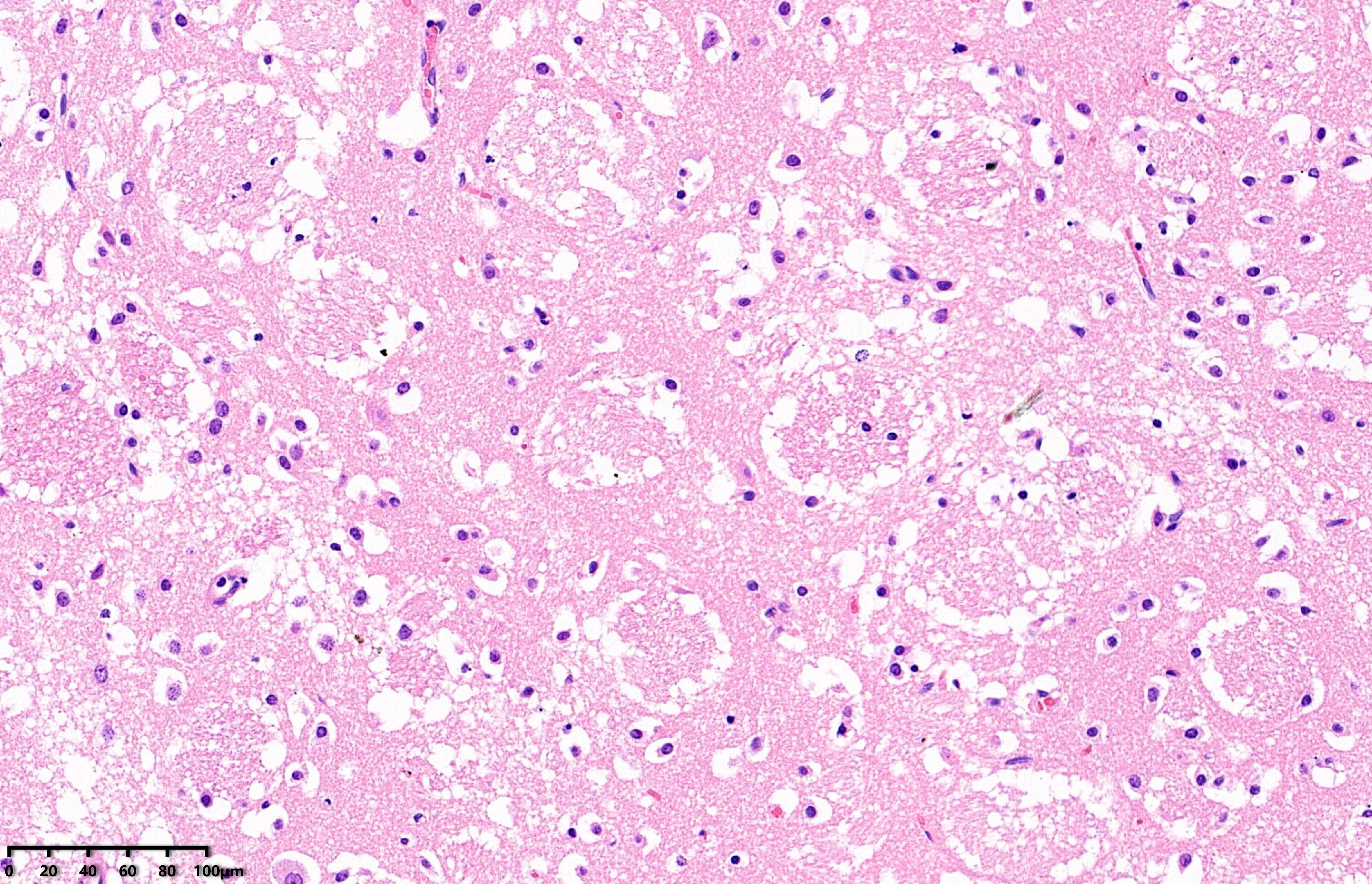

Supplement: Supplementary file 4 [file Data_Sheet_4.ZIP › Fig.4/Fig.4C-HE staining/Fig.4C-AAV-NC.jpg]

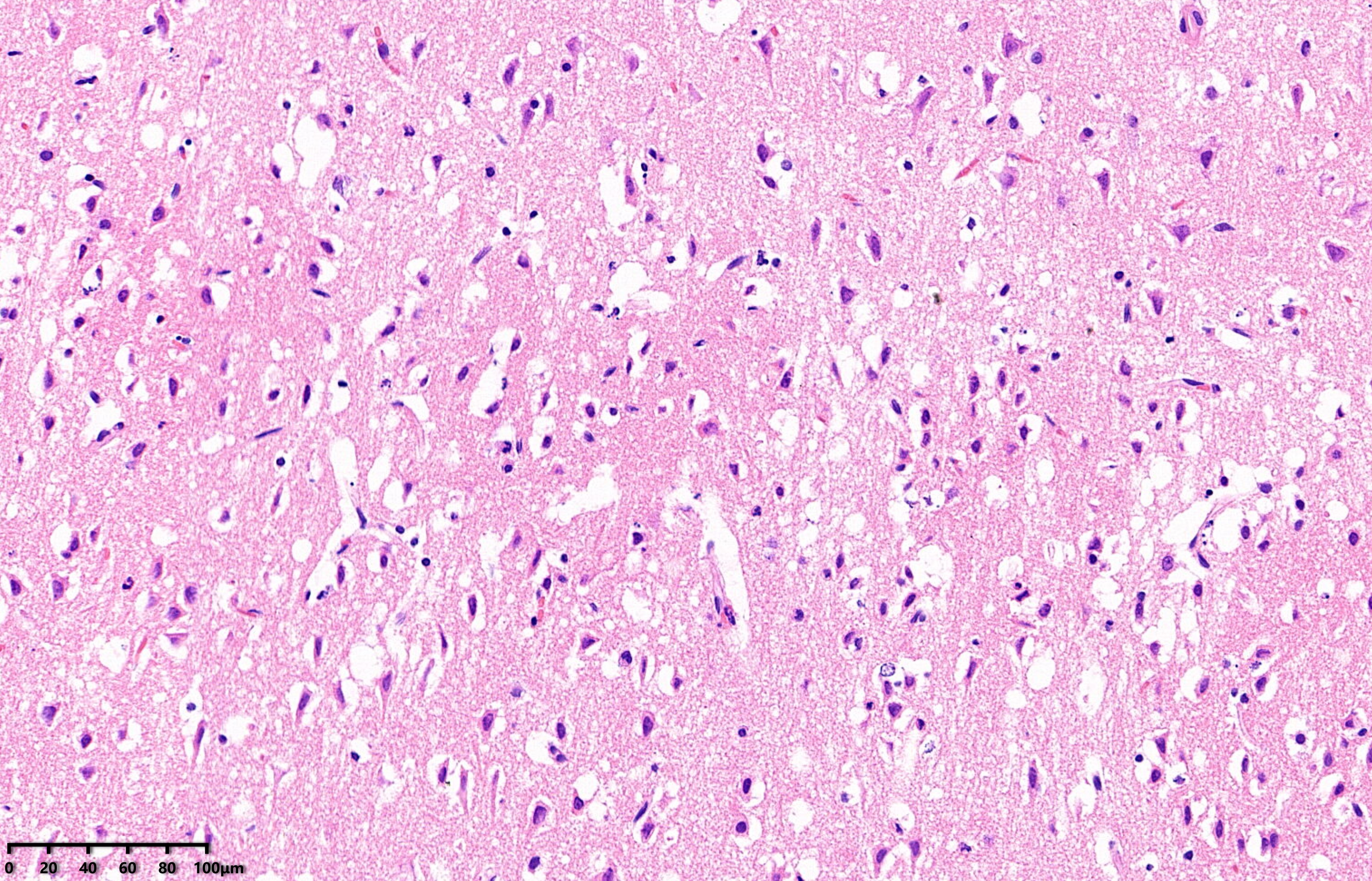

Supplement: Supplementary file 4 [file Data_Sheet_4.ZIP › Fig.4/Fig.4C-HE staining/Fig.4C-Model.jpg]

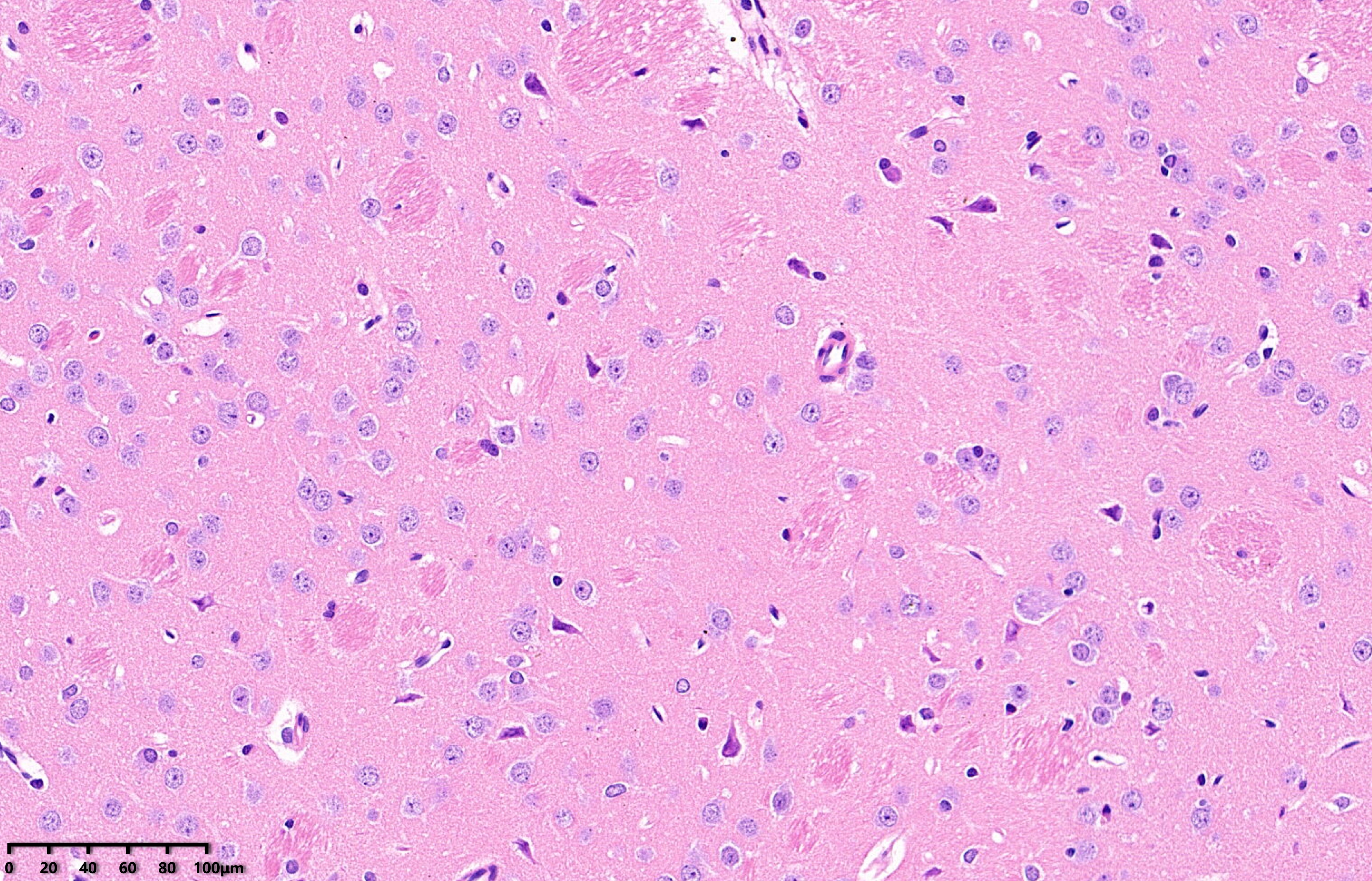

Supplement: Supplementary file 4 [file Data_Sheet_4.ZIP › Fig.4/Fig.4C-HE staining/Fig.4C-Sham.jpg]

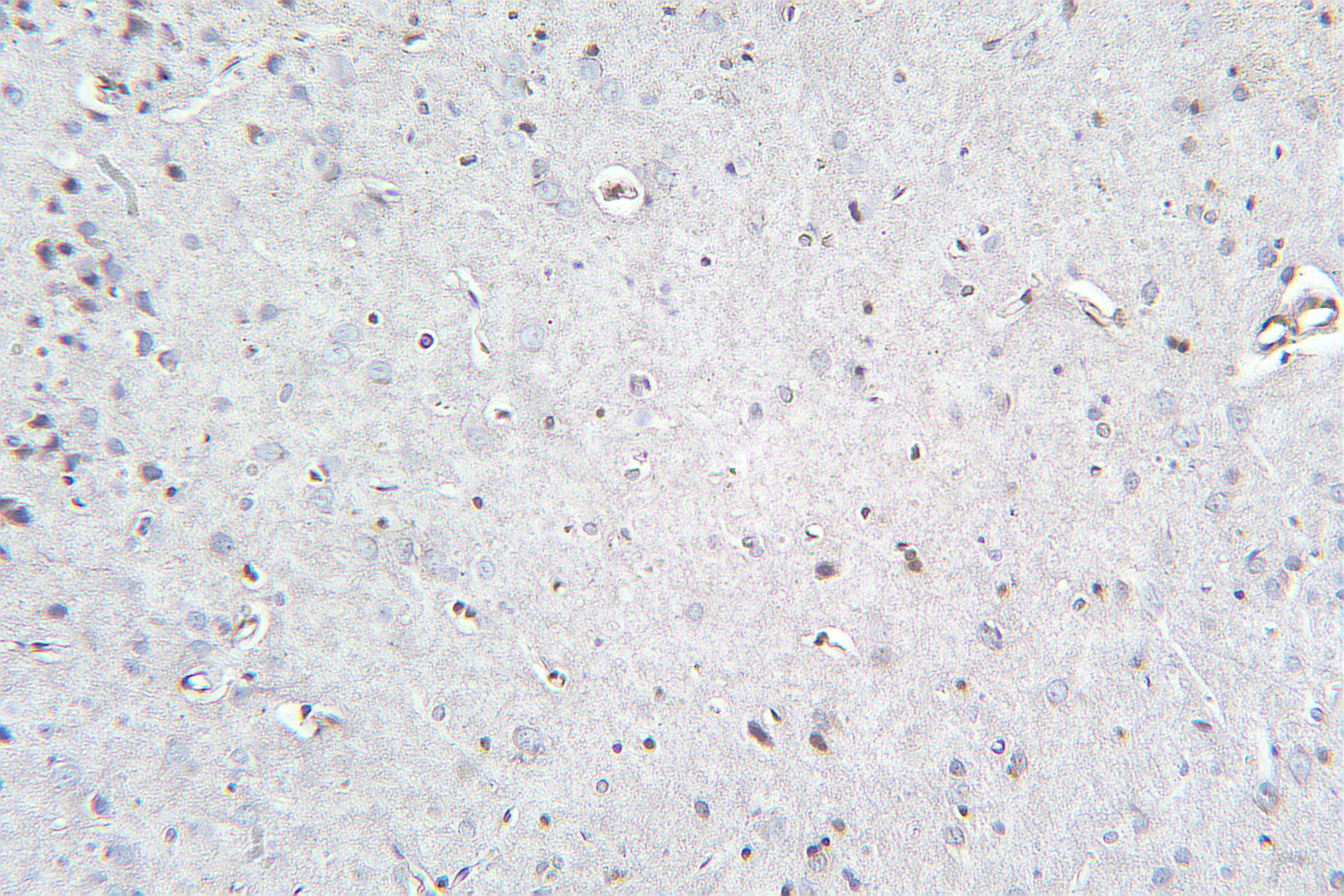

Supplement: Supplementary file 4 [file Data_Sheet_4.ZIP › Fig.4/Fig.4D-IHC staining/Fig.4D-AAV-ATF3.jpg]

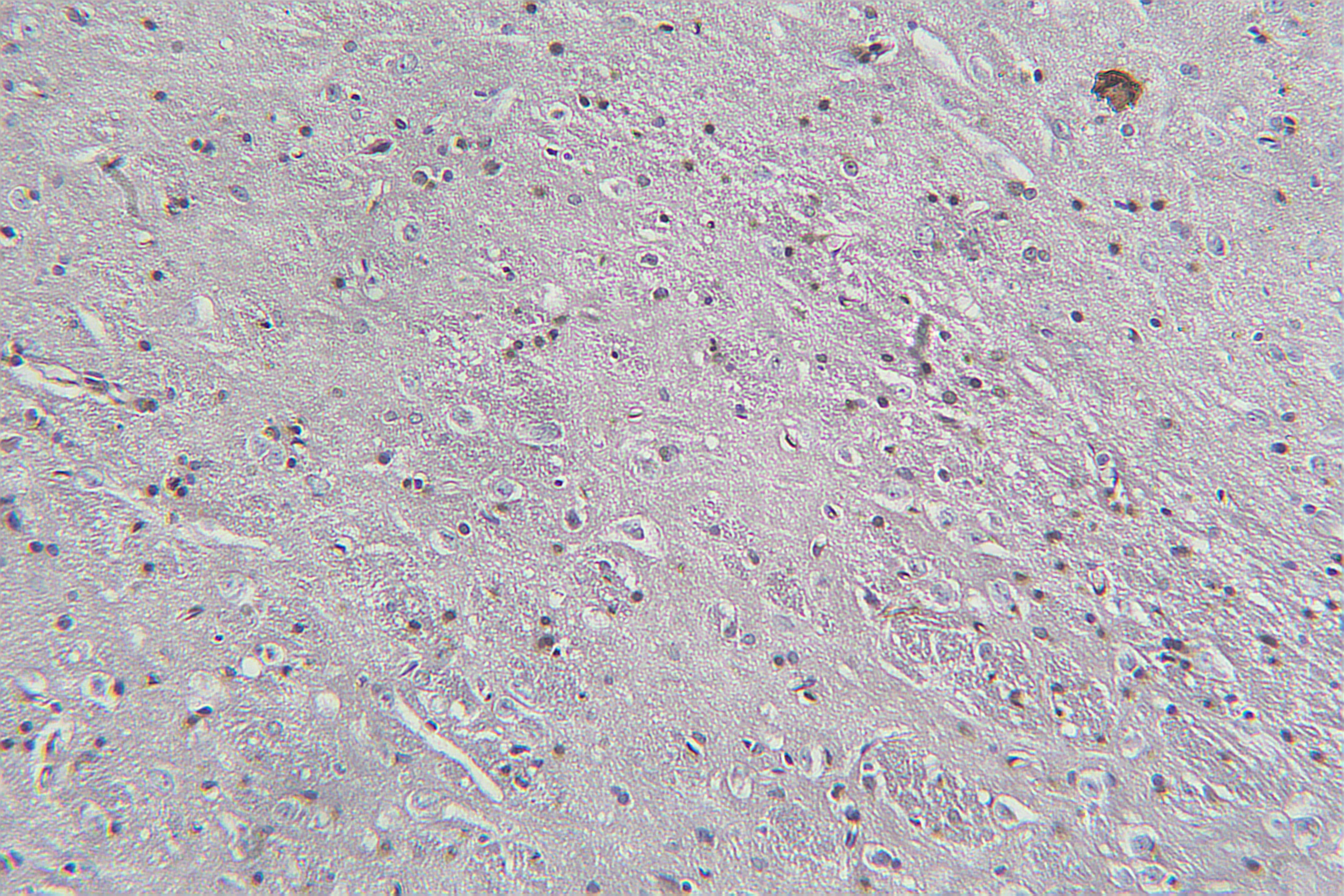

Supplement: Supplementary file 4 [file Data_Sheet_4.ZIP › Fig.4/Fig.4D-IHC staining/Fig.4D-AAV-NC.jpg]

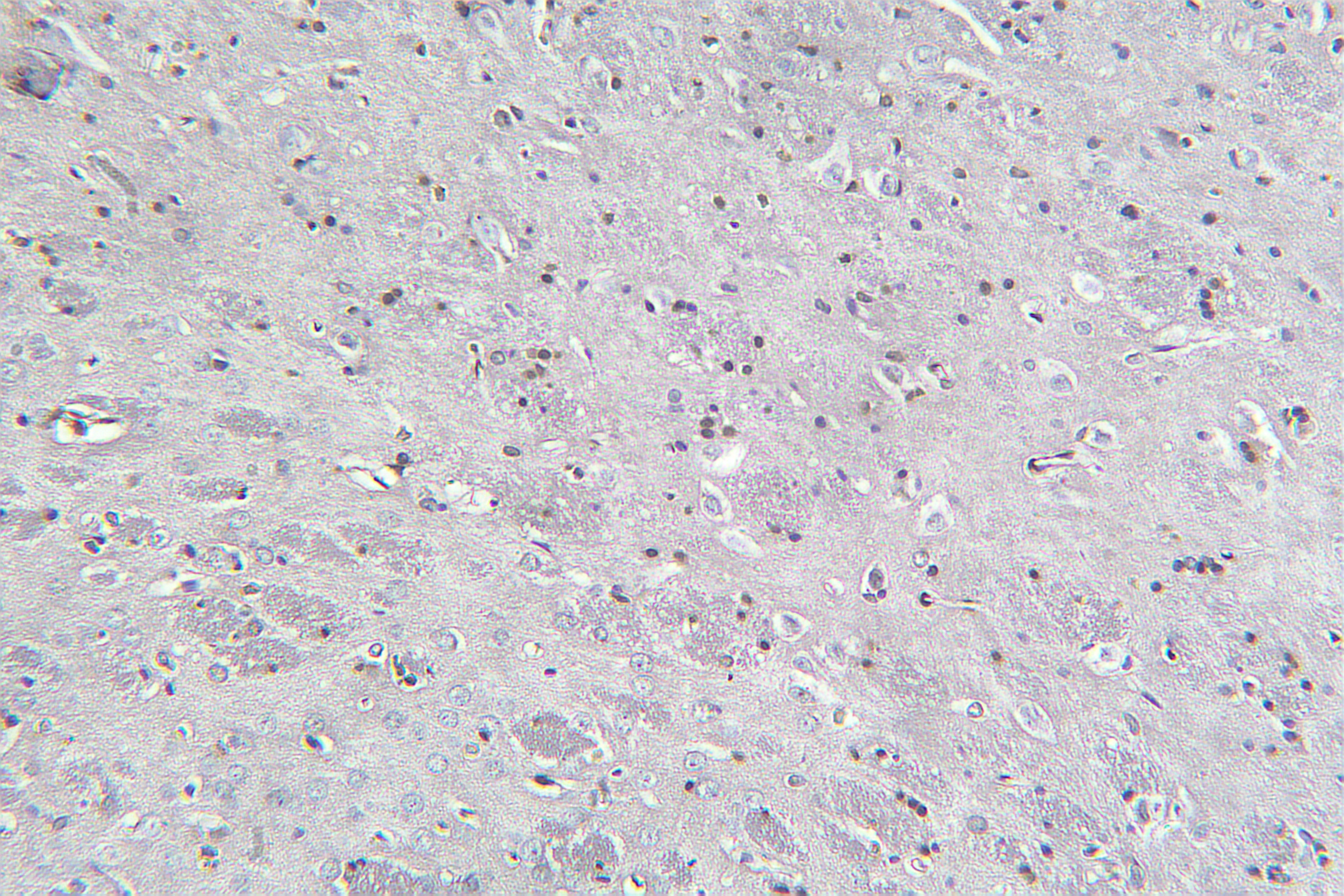

Supplement: Supplementary file 4 [file Data_Sheet_4.ZIP › Fig.4/Fig.4D-IHC staining/Fig.4D-Model.jpg]

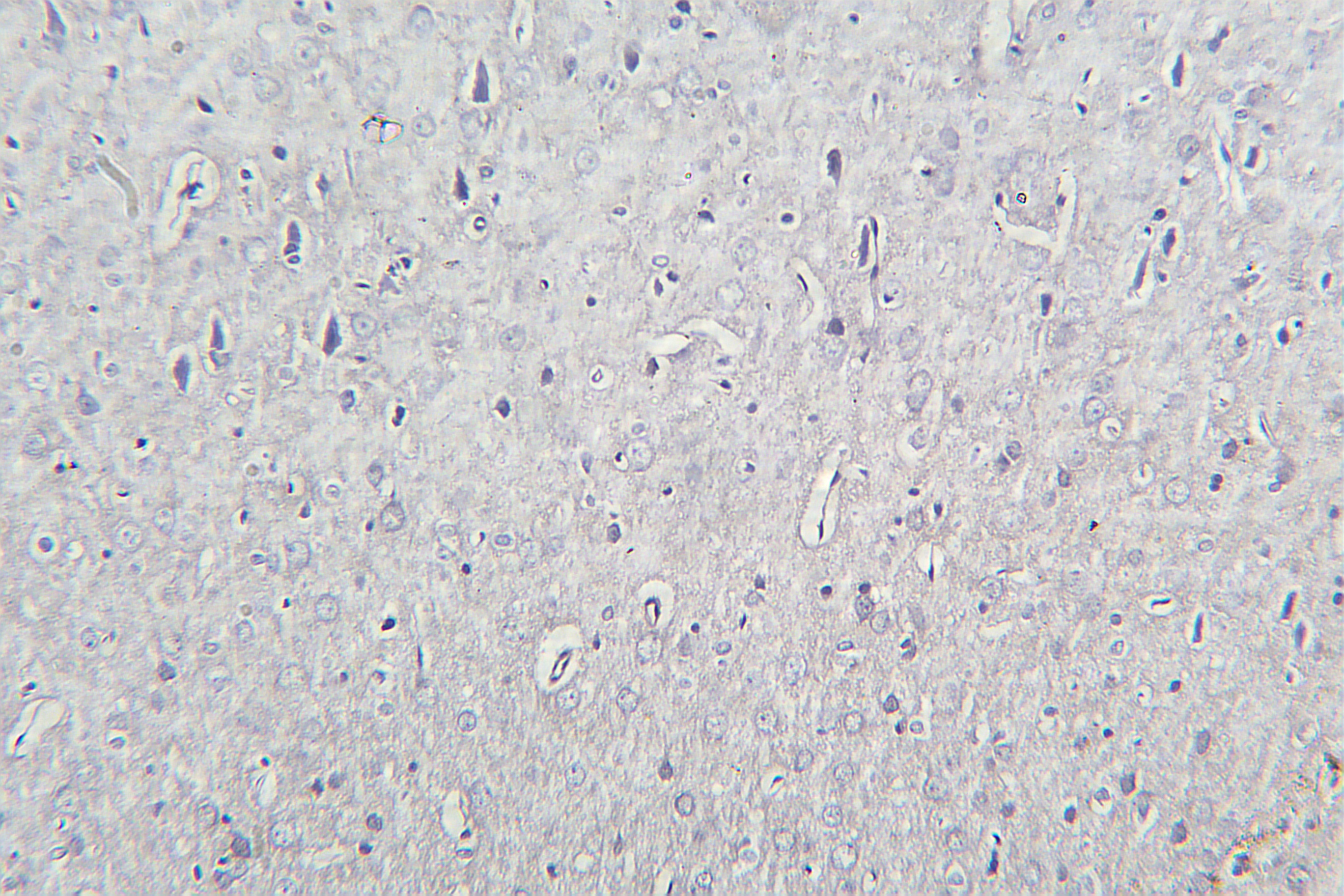

Supplement: Supplementary file 4 [file Data_Sheet_4.ZIP › Fig.4/Fig.4D-IHC staining/Fig.4D-Sham.jpg]

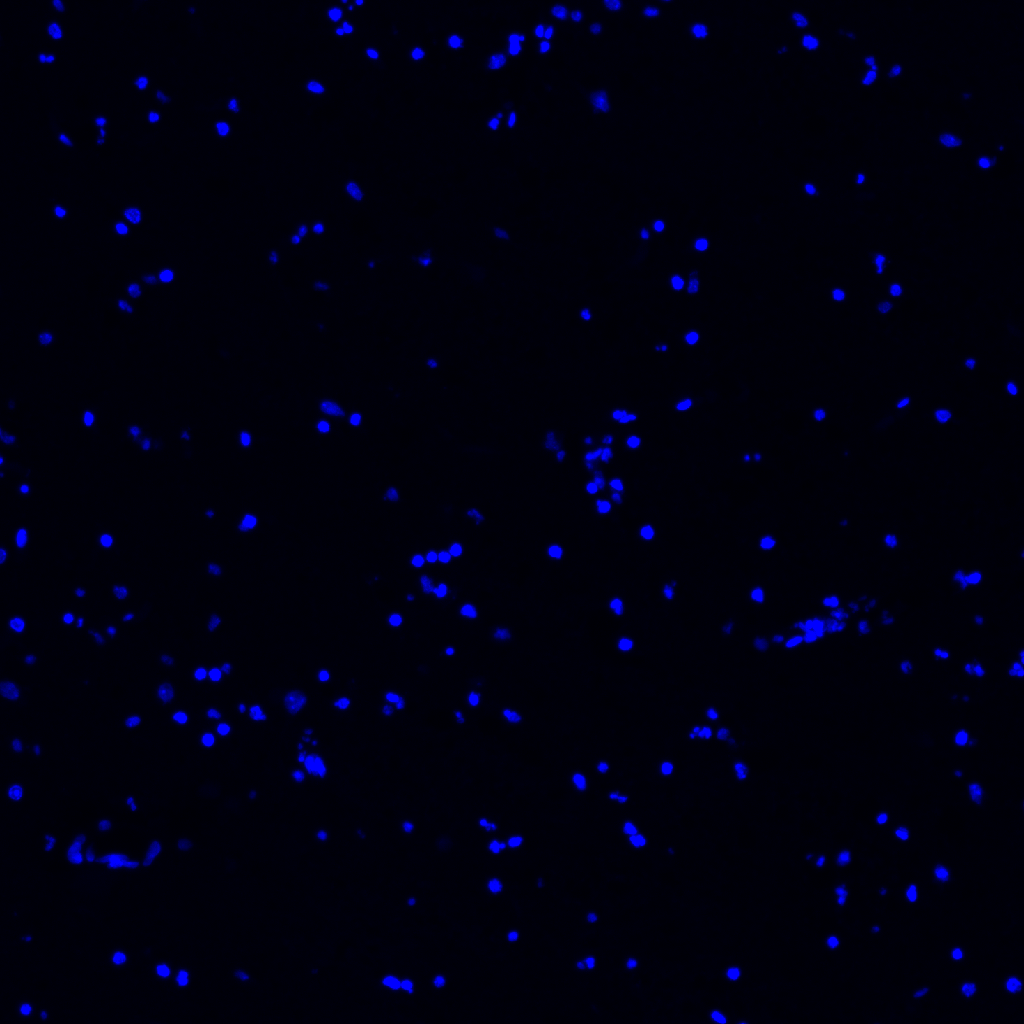

Supplement: Supplementary file 4 [file Data_Sheet_4.ZIP › Fig.4/Fig.4E-TUNEL assay/Fig.4E-AAV-ATF3-DAPI.tiff]

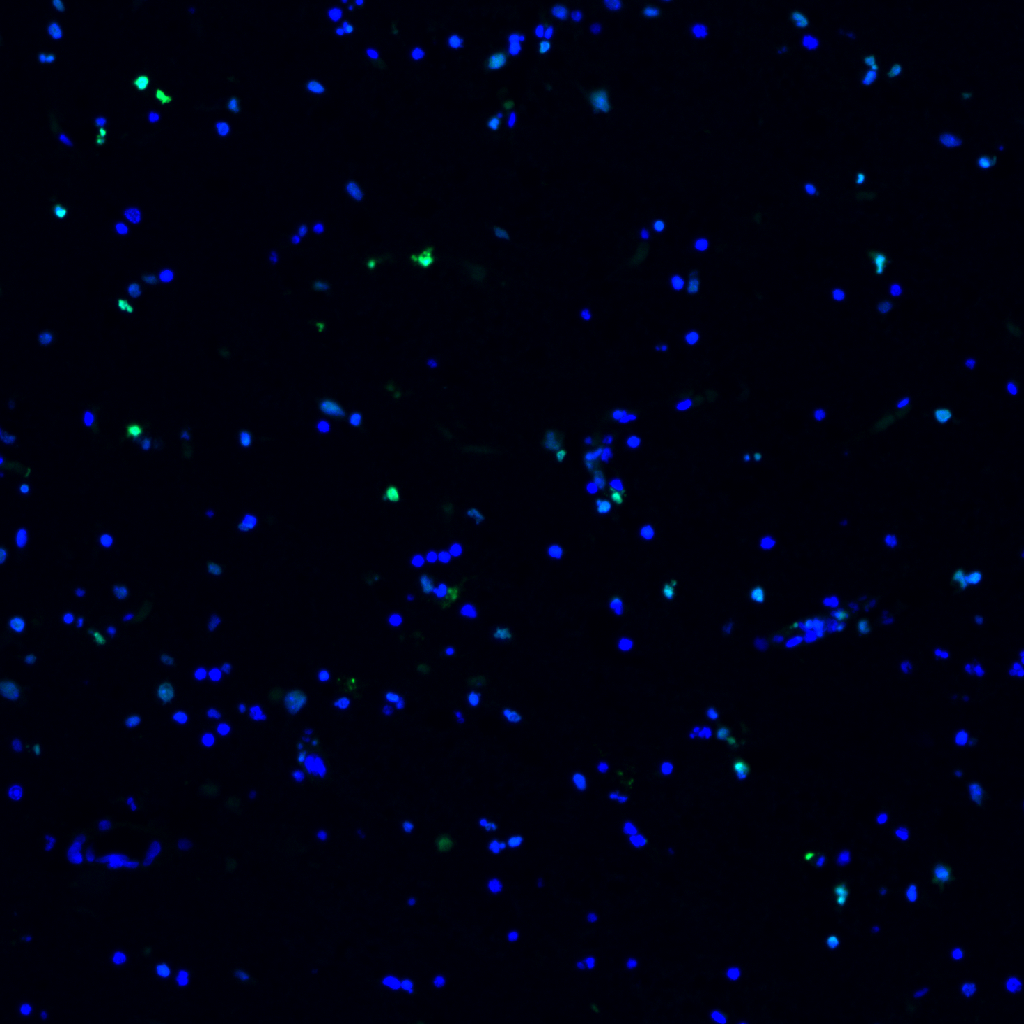

Supplement: Supplementary file 4 [file Data_Sheet_4.ZIP › Fig.4/Fig.4E-TUNEL assay/Fig.4E-AAV-ATF3-Merge.tiff]

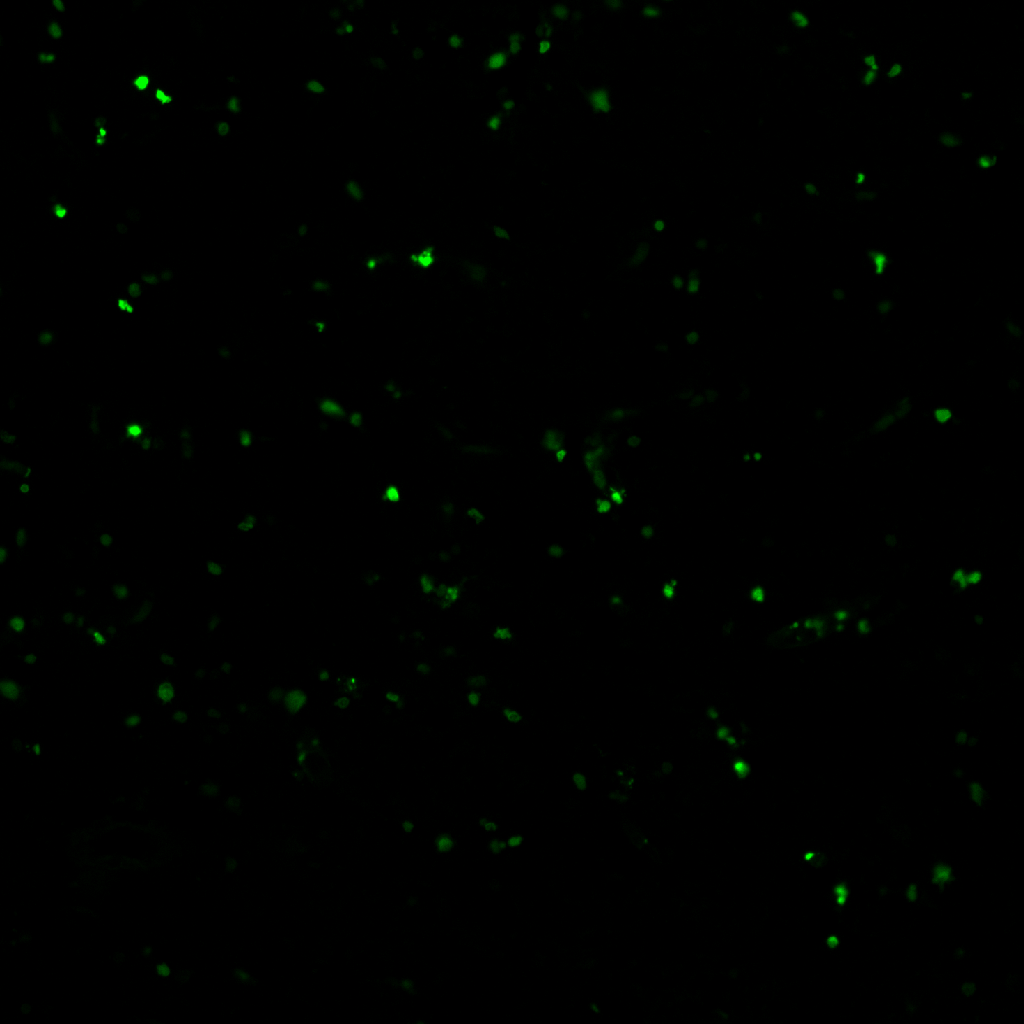

Supplement: Supplementary file 4 [file Data_Sheet_4.ZIP › Fig.4/Fig.4E-TUNEL assay/Fig.4E-AAV-ATF3-Tunel.tiff]

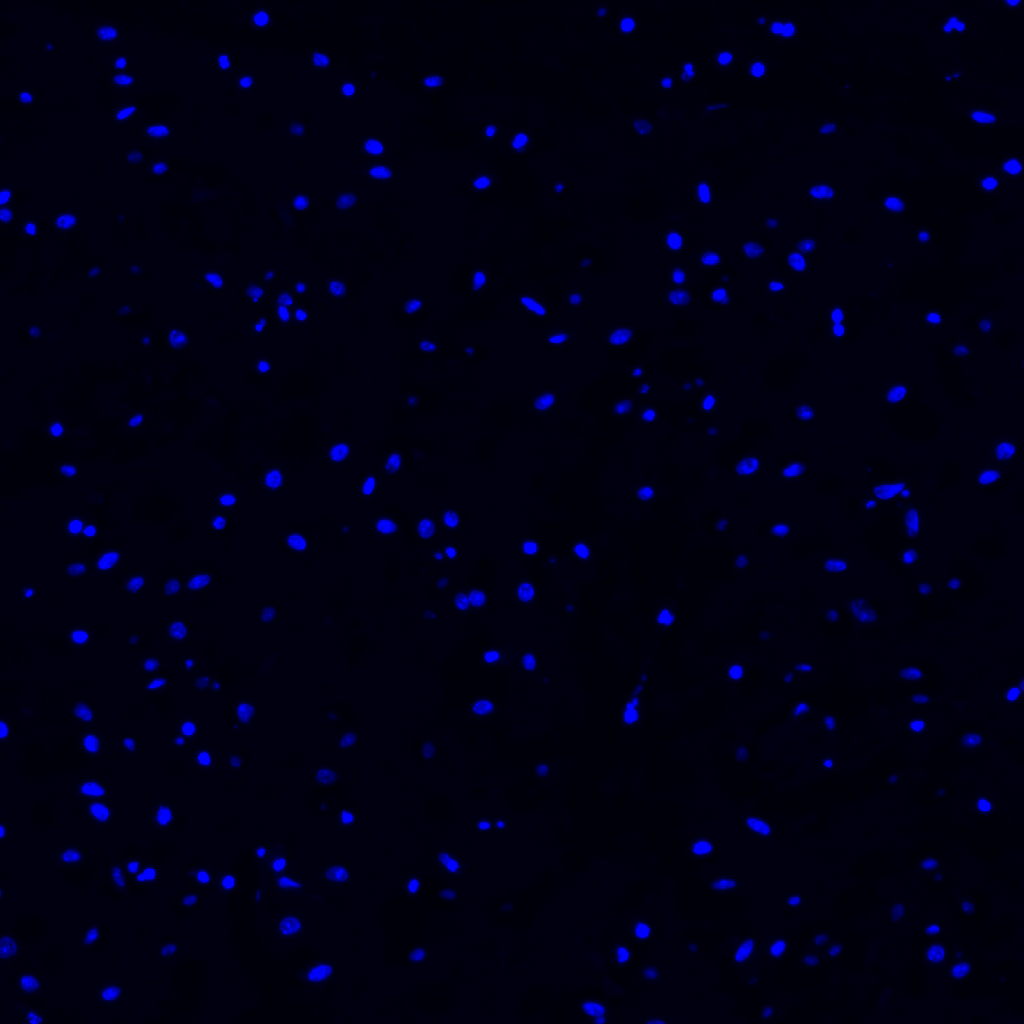

Supplement: Supplementary file 4 [file Data_Sheet_4.ZIP › Fig.4/Fig.4E-TUNEL assay/Fig.4E-AAV-NC-DAPI.tiff]

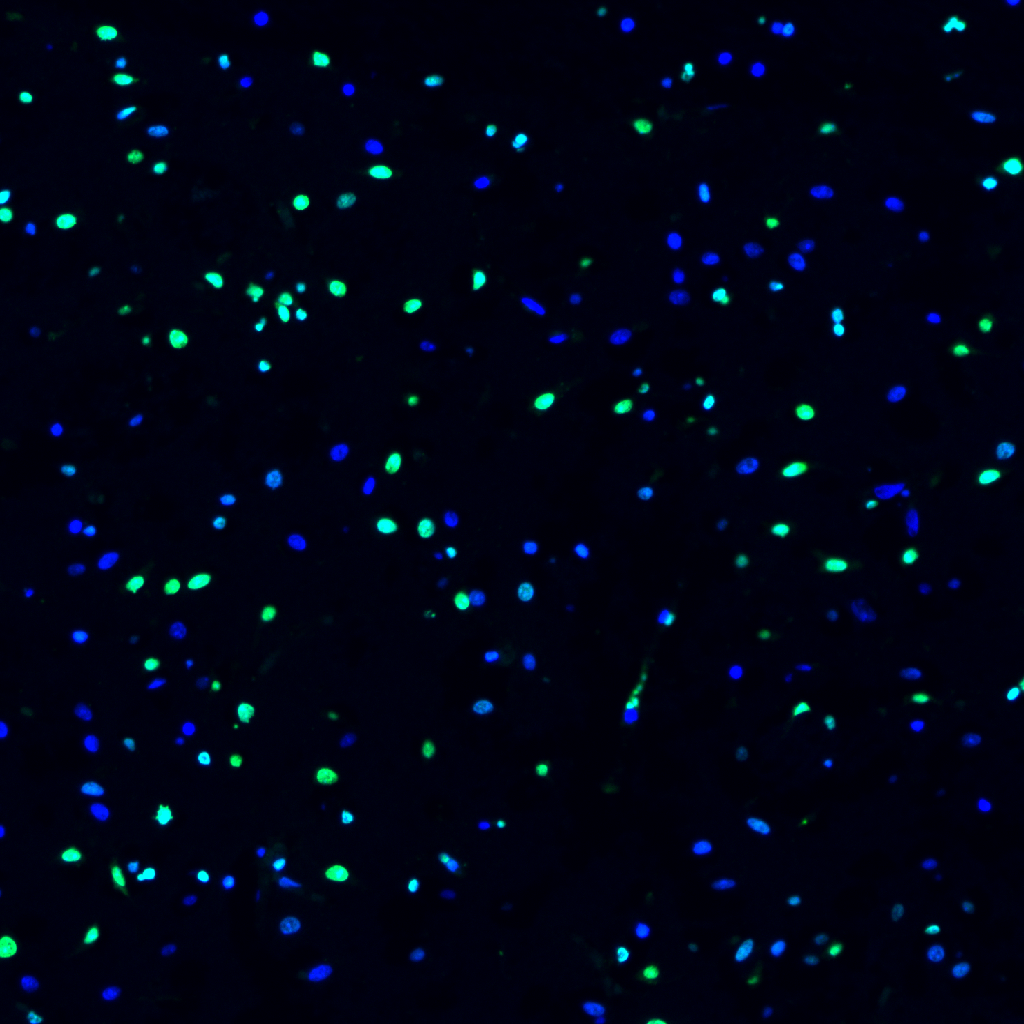

Supplement: Supplementary file 4 [file Data_Sheet_4.ZIP › Fig.4/Fig.4E-TUNEL assay/Fig.4E-AAV-NC-Merge.tiff]

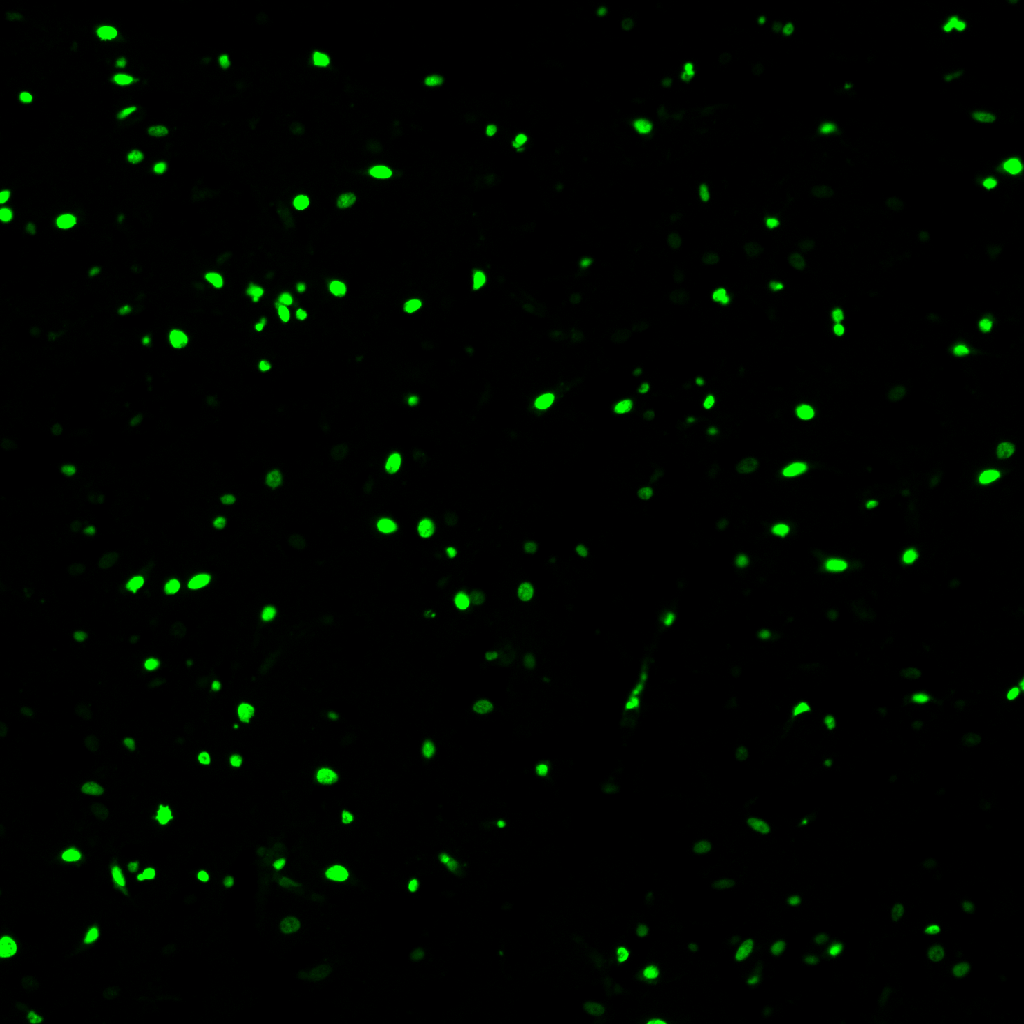

Supplement: Supplementary file 4 [file Data_Sheet_4.ZIP › Fig.4/Fig.4E-TUNEL assay/Fig.4E-AAV-NC-Tunel.tiff]

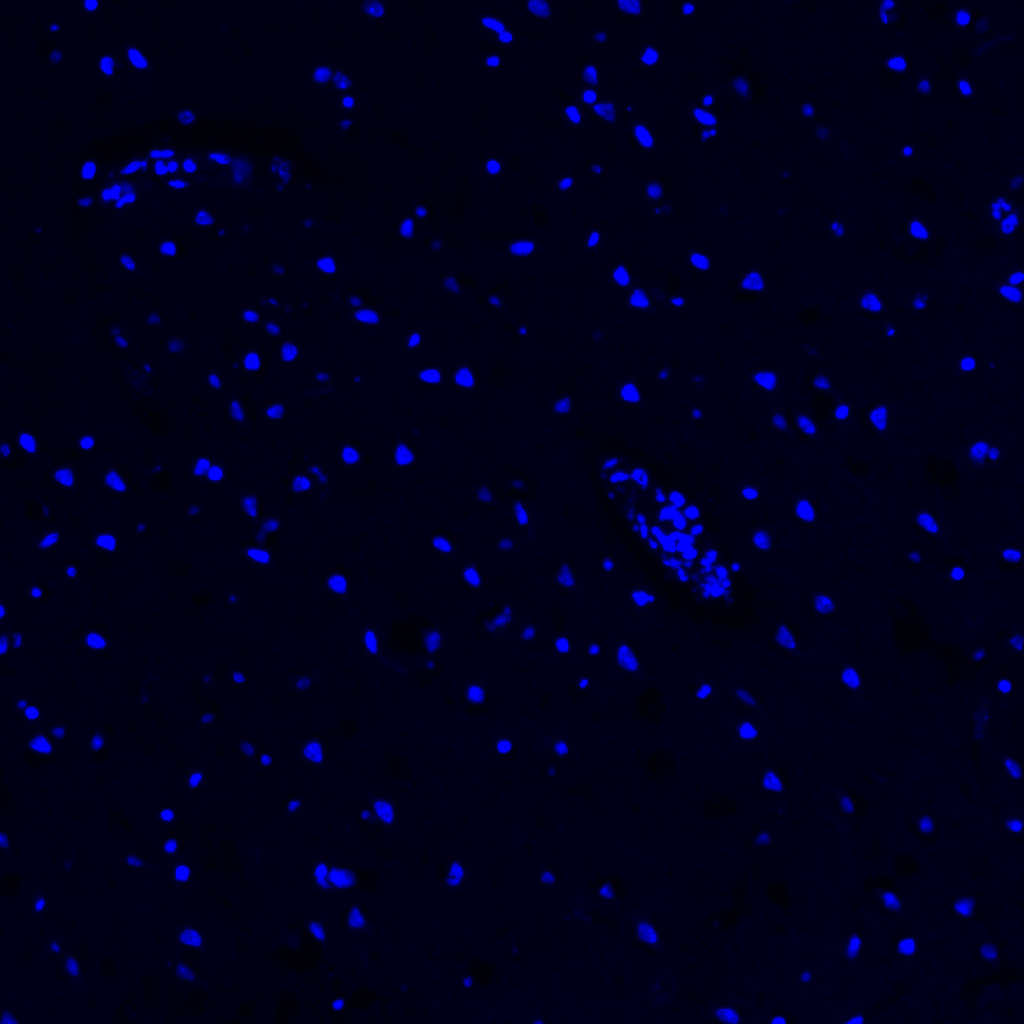

Supplement: Supplementary file 4 [file Data_Sheet_4.ZIP › Fig.4/Fig.4E-TUNEL assay/Fig.4E-Model-DAPI.tiff]

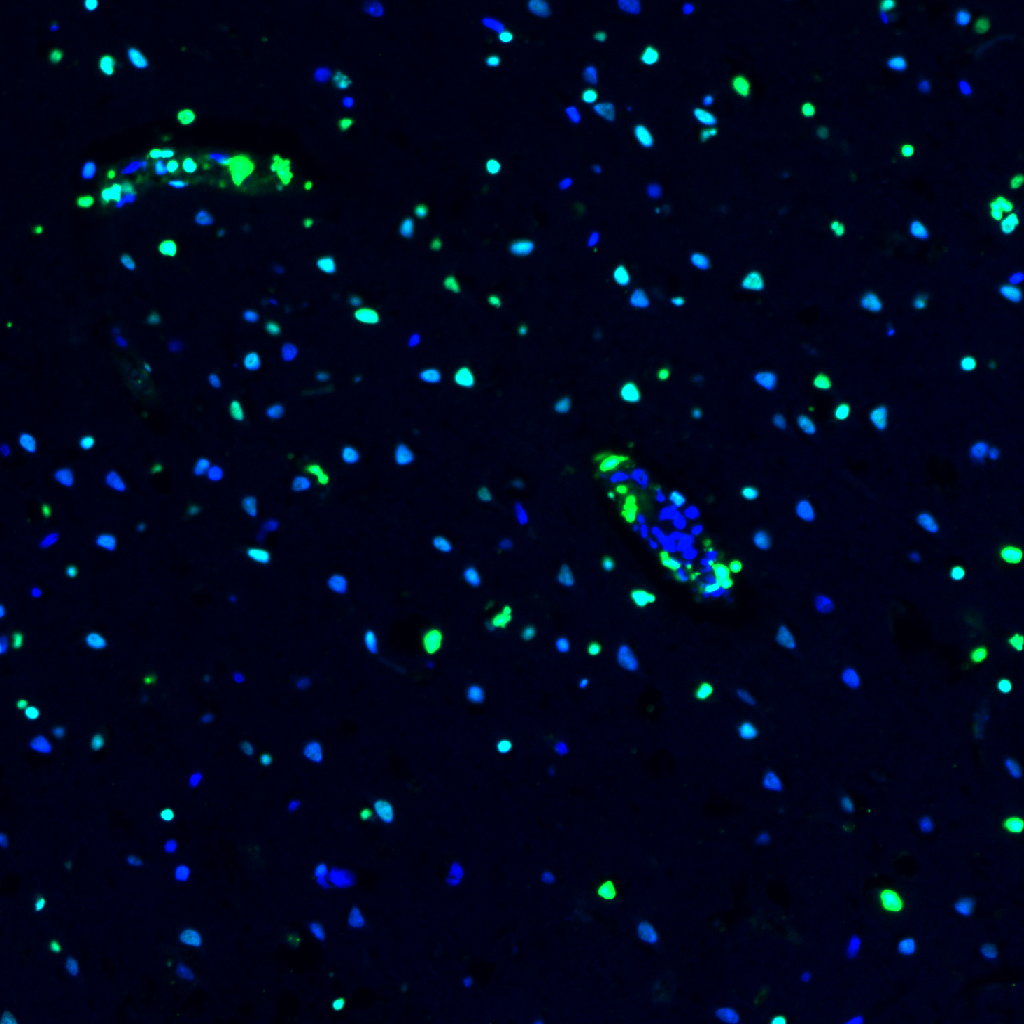

Supplement: Supplementary file 4 [file Data_Sheet_4.ZIP › Fig.4/Fig.4E-TUNEL assay/Fig.4E-Model-Merge.tiff]

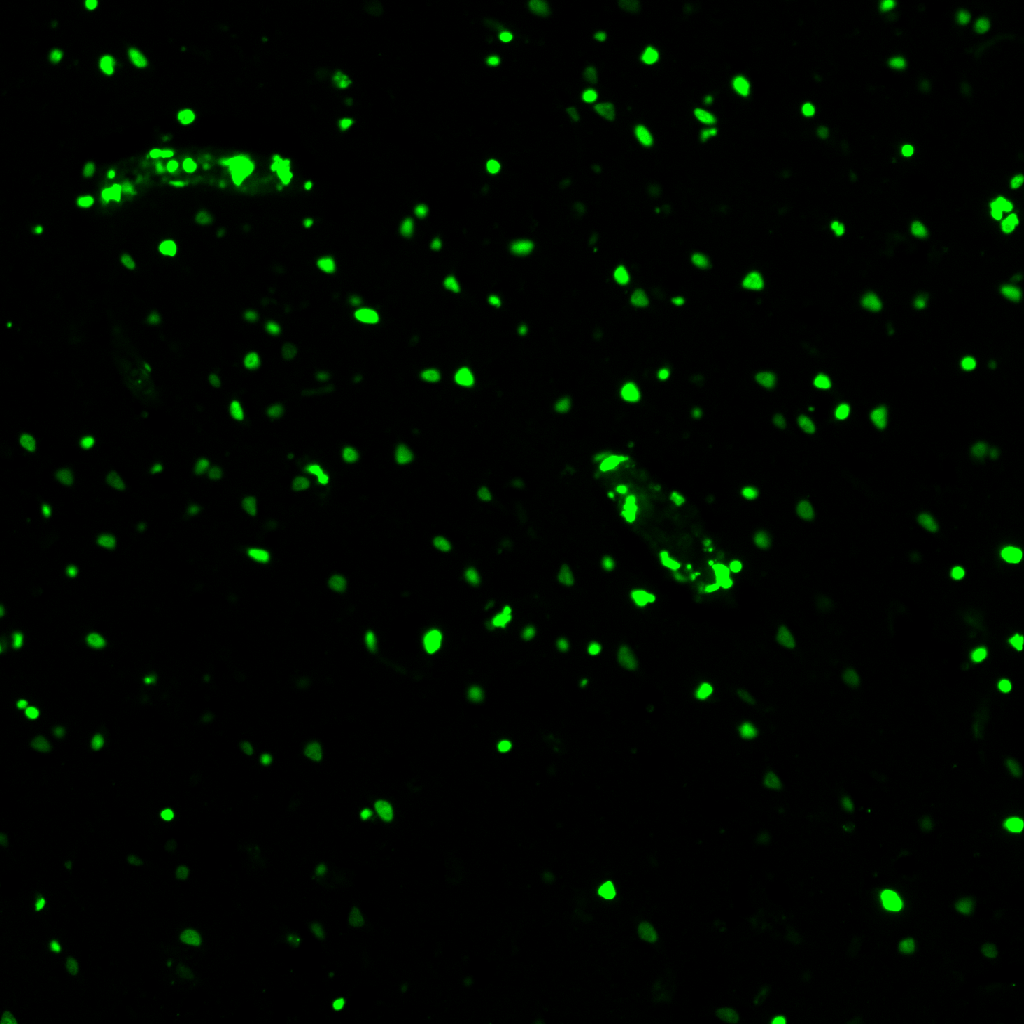

Supplement: Supplementary file 4 [file Data_Sheet_4.ZIP › Fig.4/Fig.4E-TUNEL assay/Fig.4E-Model-Tunel.tiff]

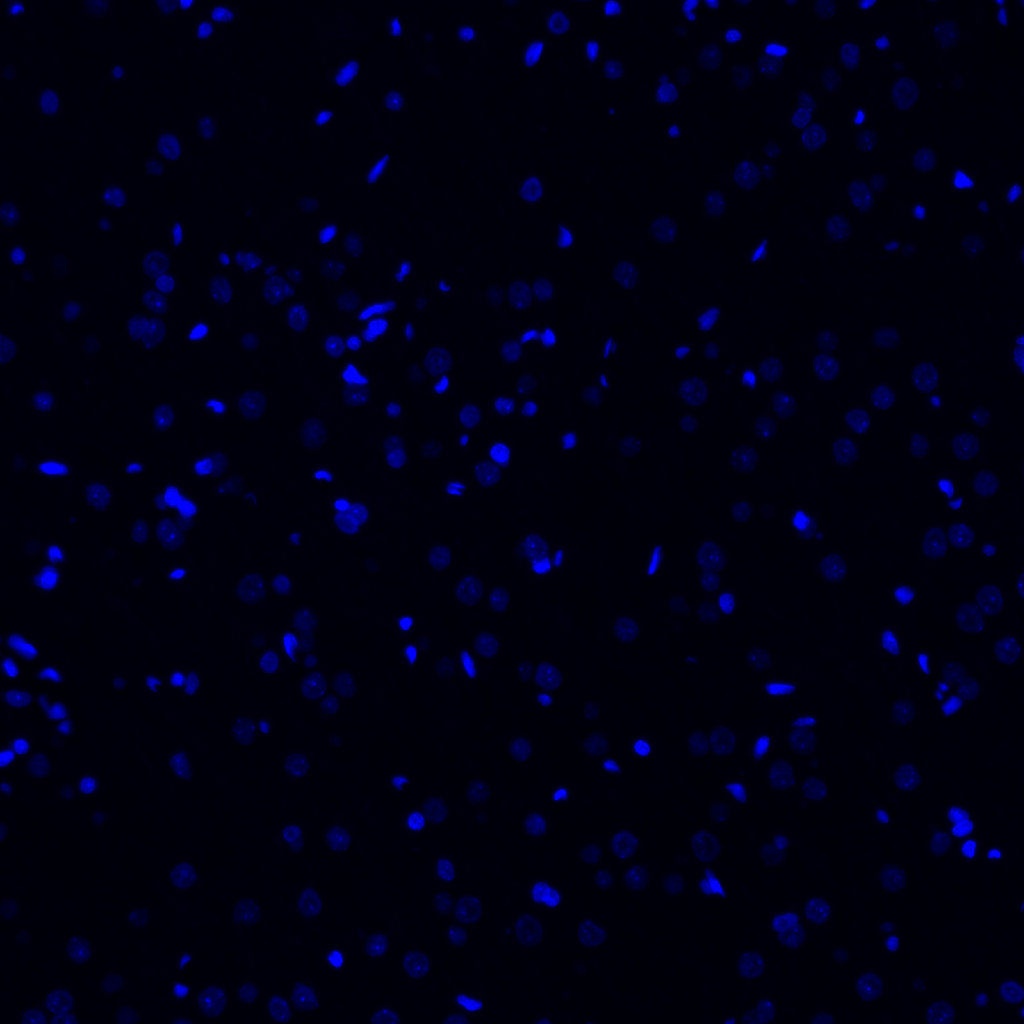

Supplement: Supplementary file 4 [file Data_Sheet_4.ZIP › Fig.4/Fig.4E-TUNEL assay/Fig.4E-Sham-DAPI.tiff]

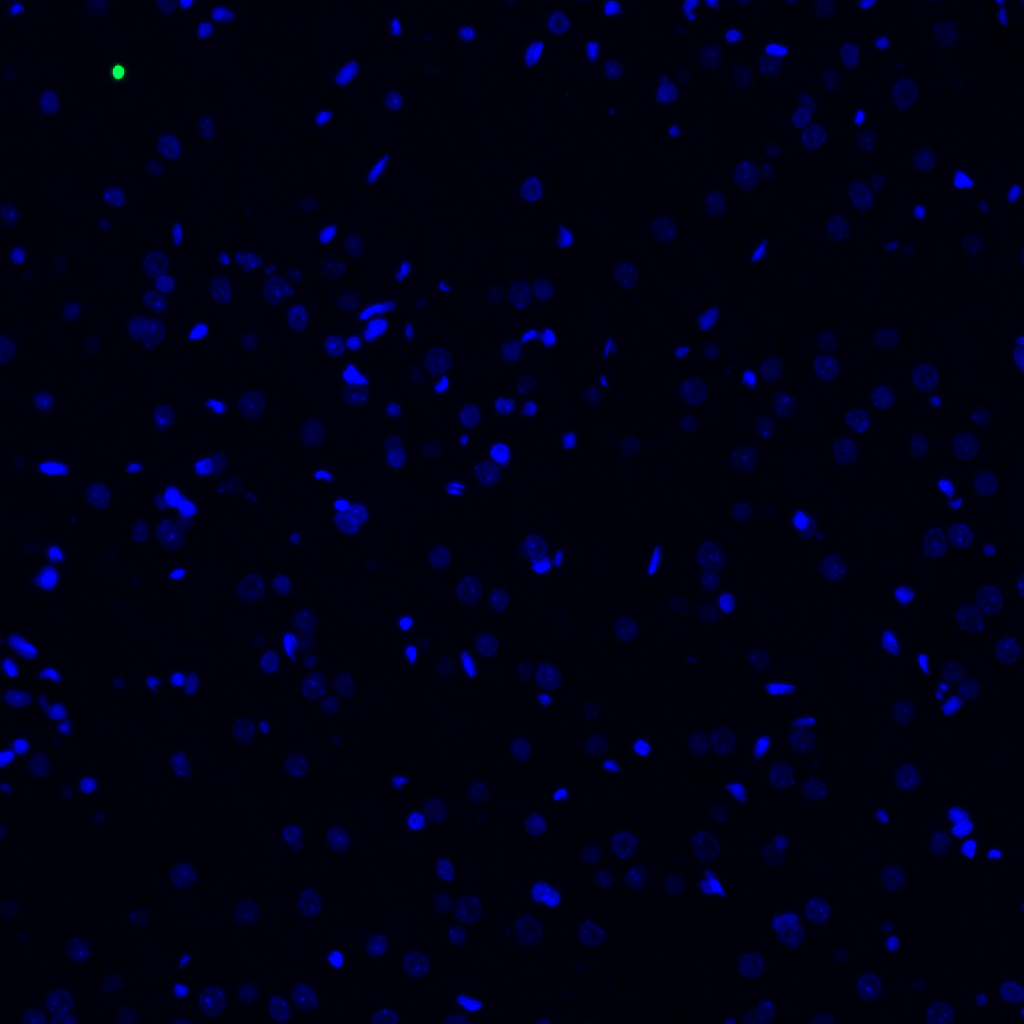

Supplement: Supplementary file 4 [file Data_Sheet_4.ZIP › Fig.4/Fig.4E-TUNEL assay/Fig.4E-Sham-Merge.tiff]

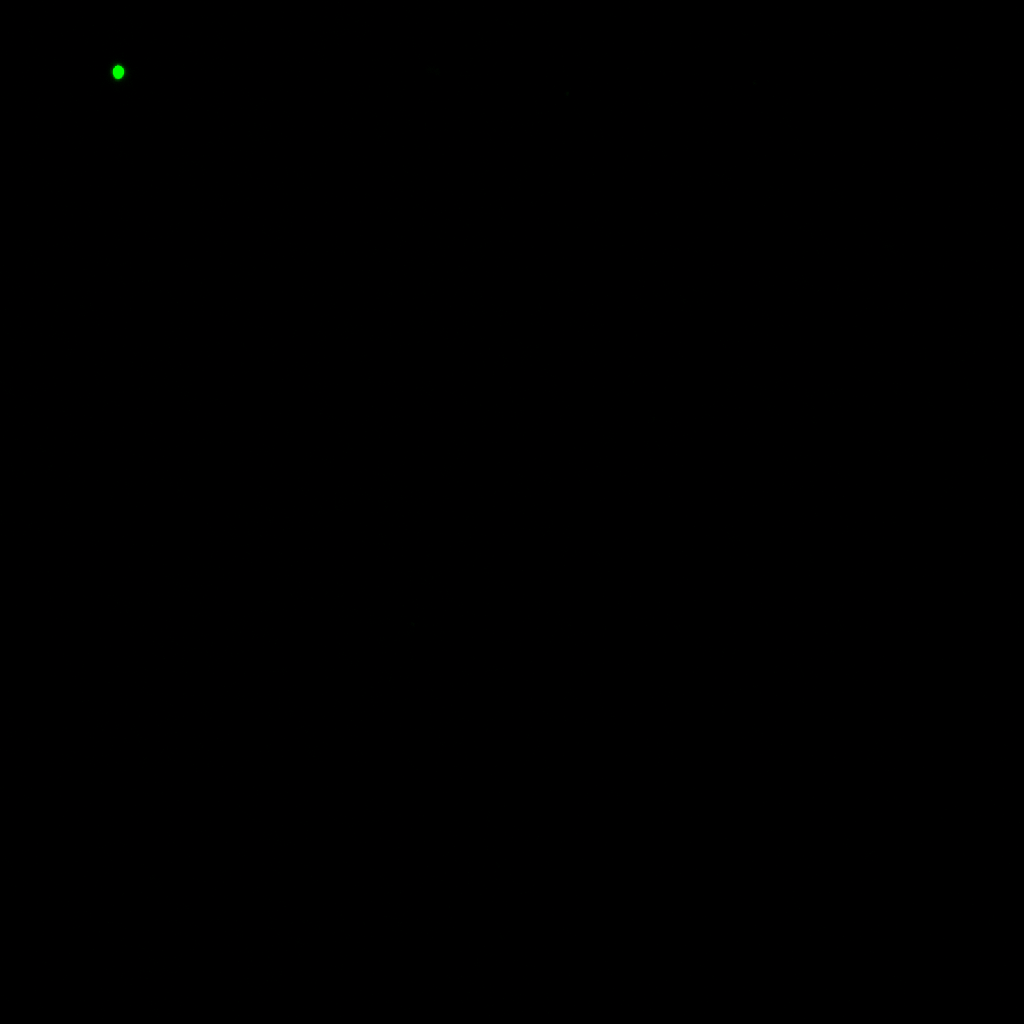

Supplement: Supplementary file 4 [file Data_Sheet_4.ZIP › Fig.4/Fig.4E-TUNEL assay/Fig.4E-Sham-Tunel.tiff]

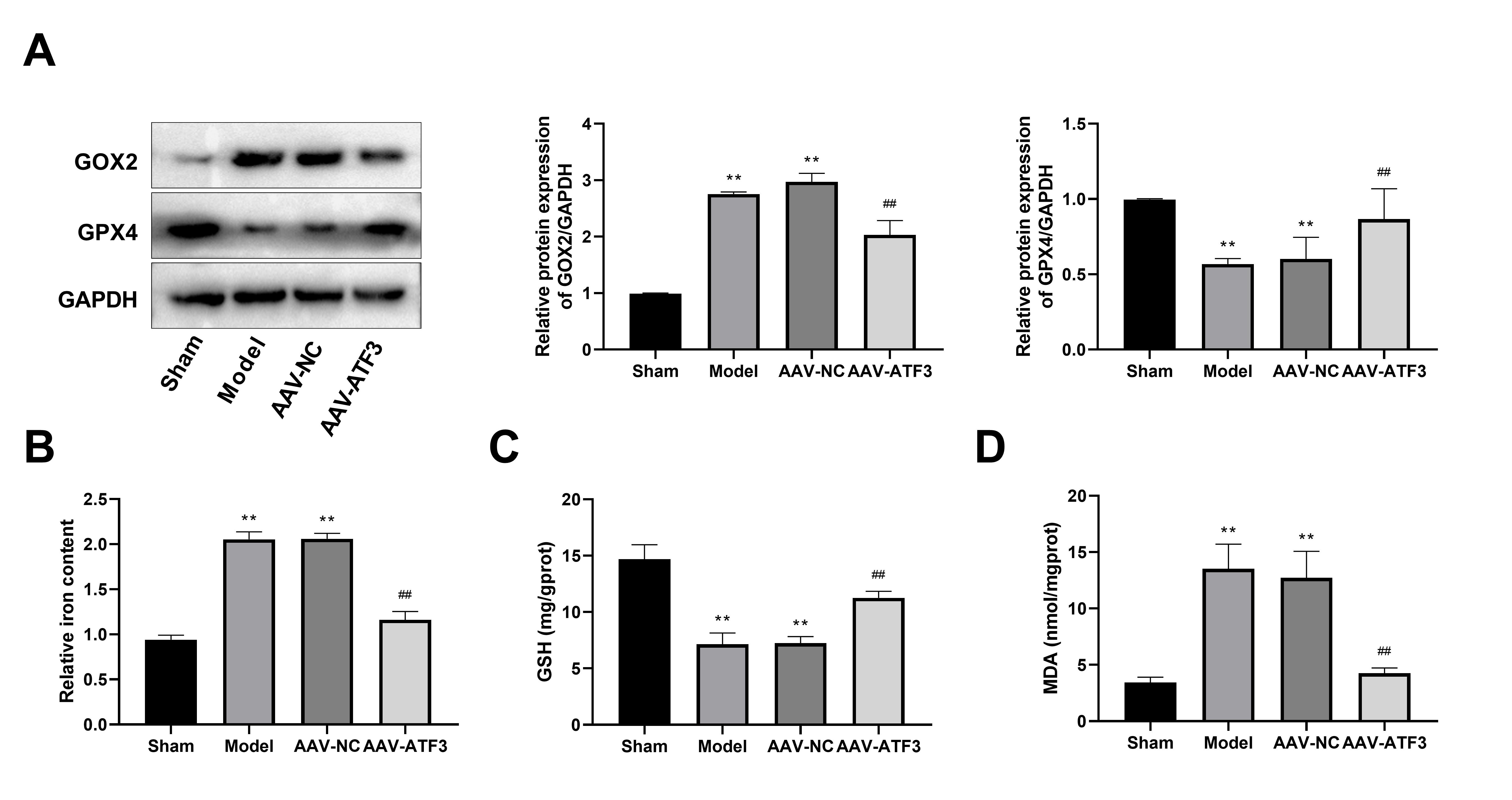

Supplement: Supplementary file 5 [file Data_Sheet_5.ZIP › Fig.5/Fig.5.jpg]

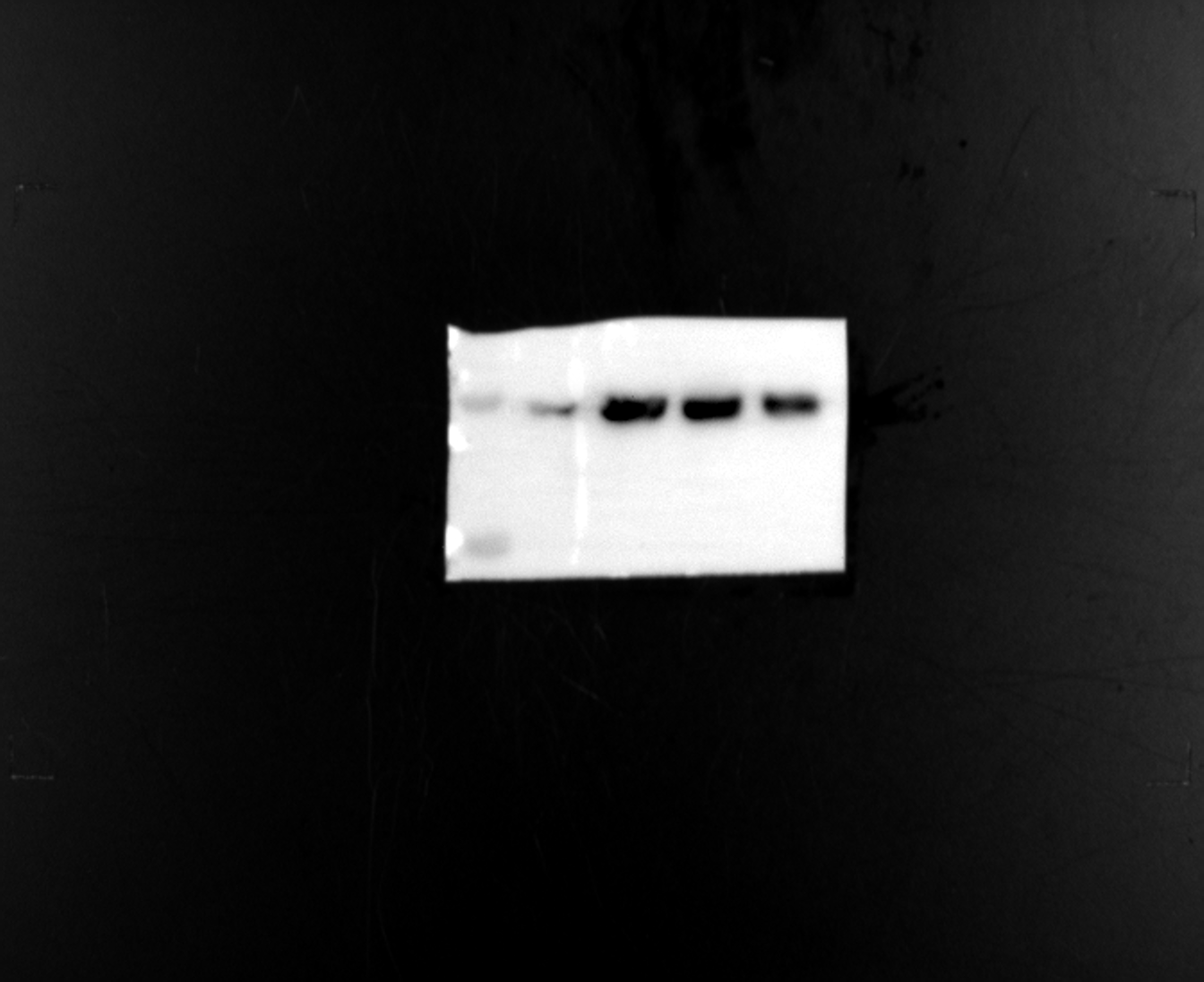

Supplement: Supplementary file 5 [file Data_Sheet_5.ZIP › Fig.5/Fig.5A-Western blot analysis/Fig.5A-COX2.Tif]

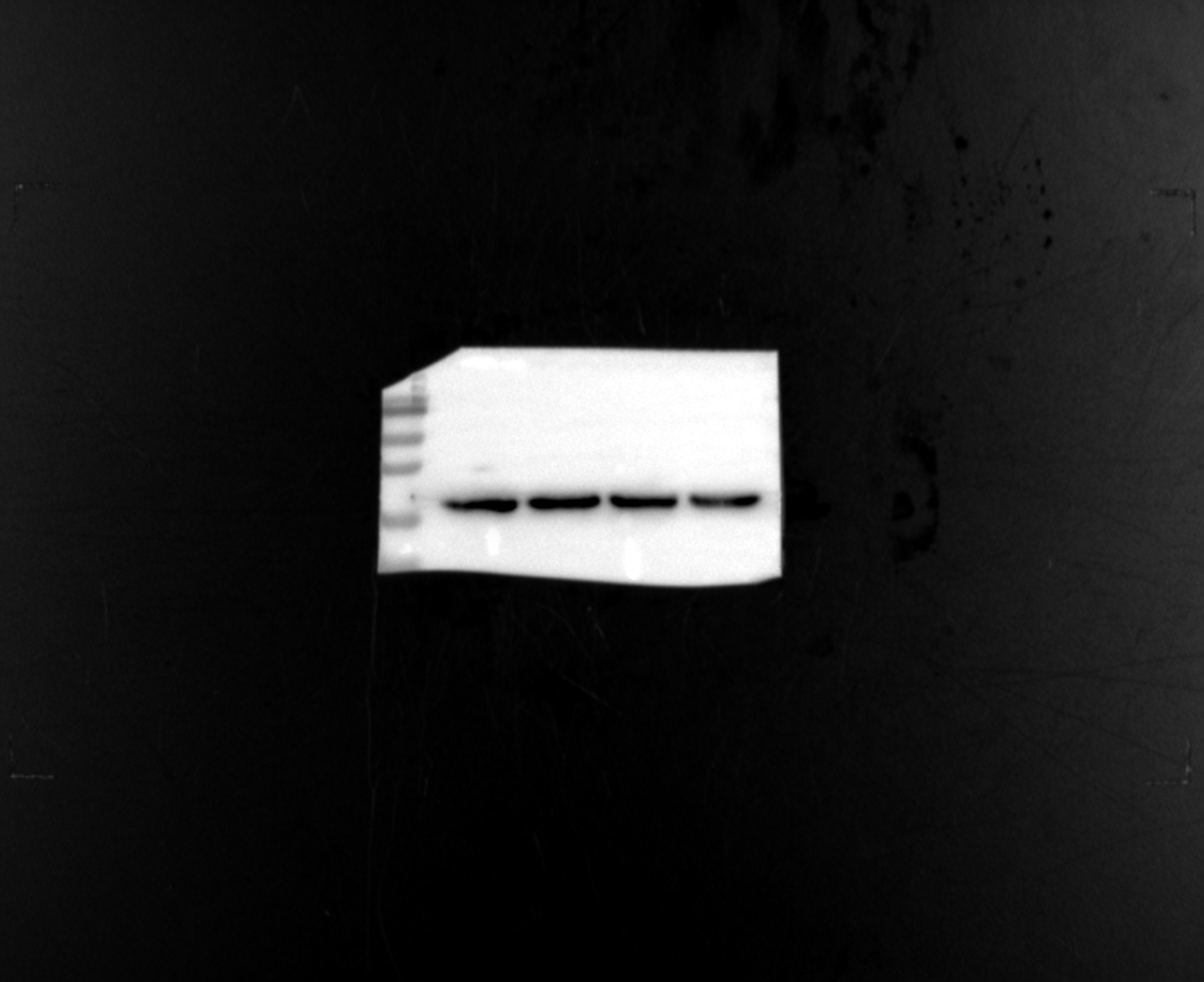

Supplement: Supplementary file 5 [file Data_Sheet_5.ZIP › Fig.5/Fig.5A-Western blot analysis/Fig.5A-GAPDH.Tif]

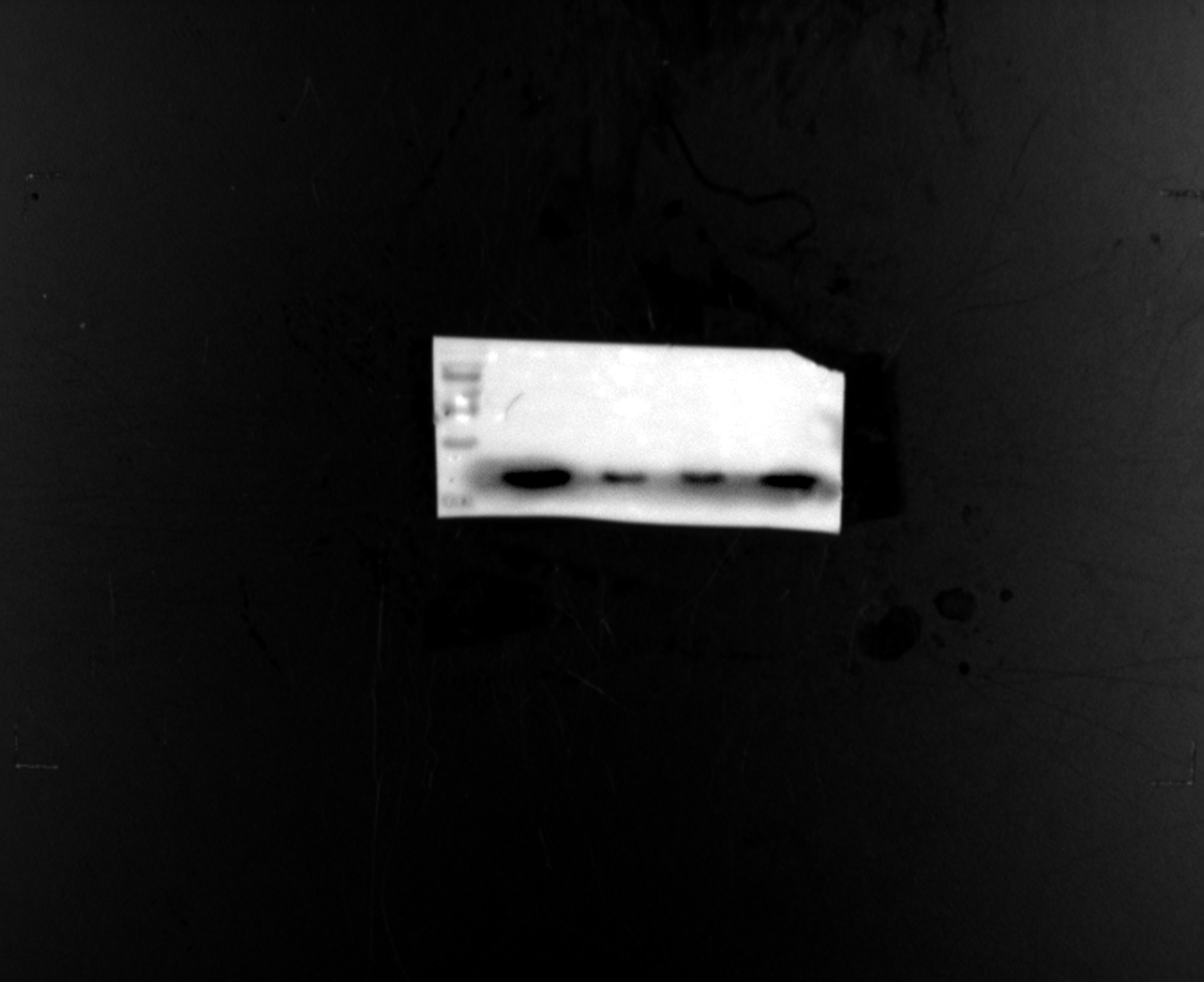

Supplement: Supplementary file 5 [file Data_Sheet_5.ZIP › Fig.5/Fig.5A-Western blot analysis/Fig.5A-GPX4.Tif]
