# Supplementary material for: Knockdown of ATF3 suppresses the progression of ischemic stroke through inhibiting ferroptosis
Source: Front Mol Neurosci. 2023 Jan 18;15:1079338. doi: 10.3389/fnmol.2022.1079338 (PMC9890179; doi:10.3389/fnmol.2022.1079338)
Supplement: Supplementary file 3 [file Data_Sheet_3.ZIP › Fig.3/Fig.3-qRT-PCR/Table S1.docx]

**Table S1.** The sequence of primers.

| **Name** | **Sequence (5’-3’)** |
| --- | --- |
| R-GAPDH-F | CTCATGACCACAGTCCATGC |
| R-GAPDH-R | TTCAGCTCTGGGATGACCTT |
| R-Hspa1b-F | CAAGATCACCATCACCAACG |
| R-Hspa1b-R | GCTGATCTTGCCCTTGAGAC |
| R-Lif-F | TCAACTGGCTCAACTCAACG |
| R-Lif-R | ACCATCCGATACAGCTCGAC |
| R-Tfpi2-F | ATCCCATCATTTTGCTCCAG |
| R-Tfpi2-R | TCGGCAGGAAATCACCTATC |
| R-Ptx3-F | TCTCTGGTCTGCAGTGTTGG |
| R-Ptx3-R | GTCAGTGGCCTGTAACAGCA |
| R-Atf3-F | CAGAGCCTGGTGTTGTGCTA |
| R-Atf3-R | TCCCAGCTGAAATGCTCTGG |
| R-Smyd1-F | CAAAGGCAGAGGACTGAAGG |
| R-Smyd1-R | ATGGCAGAGCACTCGTTCTT |
| R-Tacr2-F | ATTGCTGCCTTAACCACAGG |
| R-Tacr2-R | CCCCGTCATGAACAAAGTCT |
